# A Biophysical Model of CRISPR/Cas9 Activity for Rational Design of Genome Editing and Gene Regulation

**DOI:** 10.1371/journal.pcbi.1004724

**Published:** 2016-01-29

**Authors:** Iman Farasat, Howard M. Salis

**Affiliations:** 1 Department of Chemical Engineering, Pennsylvania State University, University Park, Pennsylvania, United States of America; 2 Department of Biological Engineering, Pennsylvania State University, University Park, Pennsylvania, United States of America; Princeton University, UNITED STATES

## Abstract

The ability to precisely modify genomes and regulate specific genes will greatly accelerate several medical and engineering applications. The CRISPR/Cas9 (Type II) system binds and cuts DNA using guide RNAs, though the variables that control its on-target and off-target activity remain poorly characterized. Here, we develop and parameterize a system-wide biophysical model of Cas9-based genome editing and gene regulation to predict how changing guide RNA sequences, DNA superhelical densities, Cas9 and crRNA expression levels, organisms and growth conditions, and experimental conditions collectively control the dynamics of dCas9-based binding and Cas9-based cleavage at all DNA sites with both canonical and non-canonical PAMs. We combine statistical thermodynamics and kinetics to model Cas9:crRNA complex formation, diffusion, site selection, reversible R-loop formation, and cleavage, using large amounts of structural, biochemical, expression, and next-generation sequencing data to determine kinetic parameters and develop free energy models. Our results identify DNA supercoiling as a novel mechanism controlling Cas9 binding. Using the model, we predict Cas9 off-target binding frequencies across the lambdaphage and human genomes, and explain why Cas9’s off-target activity can be so high. With this improved understanding, we propose several rules for designing experiments for minimizing off-target activity. We also discuss the implications for engineering dCas9-based genetic circuits.

## Author Summary

The CRISPR/Cas9 immunity system has the potential to revolutionize medicine and biotechnology by enabling researchers to cut an organism’s genomic DNA at precise locations. While Cas9 is perhaps the most versatile and easy-to-use technique for gene therapy developed yet, it is not perfect; the enzyme can also cut DNA at unwanted locations in an organism’s genome. Cas9’s off-target activity must be greatly minimized to further improve its utility. Here, we develop a system-wide, quantitative, physical model to better understand all the factors that collectively control Cas9’s off-target cleavage. We solve for the unknown parameters using gene regulation data from our laboratory as well as structural, biochemical, and next-generation sequencing data from other laboratories. Using the model in several examples, we explain how Cas9 identifies on-target versus off-target DNA sites, depending on the guide RNA sequence, the Cas9 and crRNA expression levels, the organism’s genome, and the organism’s cellular growth rate. We then propose several rules for designing experiments with minimal off-target activity.

## Introduction

The RNA-mediated Cas9 adaptive immunity system (CRISPR type II) has revolutionized genome engineering by enabling the precision cutting of DNA that can be customized to target any sequence [1,2,3,4,5,6], while being functional in a broad range of prokaryotes and eukaryotes, including bacteria, yeast, flies, fish, plants, worms, monkeys, mice, rats, rabbits, frogs, and human cell lines [3,7,8,9,10,11,12,13,14,15,16,17,18]. By forcing the host to repair these precision DNA cuts, the CRISPR/Cas9 system allows recombinant DNA to be inserted at desired genome locations, and therefore can be used for performing high-throughput gene knockouts, loss-of-function screening, artificial immunization, removal of latent genome-encoded viruses, and site-specific gene therapy applications [19,20,21,22]. A nuclease-deficient version of Cas9, called dCas9, retains its RNA-guided DNA binding activity and has been used as a transcription factor to tightly control gene expression levels and rewire a host's transcriptional regulatory network [23]. Multiple dCas9-based repression and activation devices, including within layered genetic circuits, have been developed in bacteria, yeast, and mammalian cells; these genetic circuits can regulate a targeted promoter's transcription rate by up to 1000-fold [5,24,25,26,27]. In principle, the expression of multiple guide RNAs, working with dCas9, enables the regulation of many promoters simultaneously, and provides an almost limitless source of programmable transcription factors.

Based on recent observations, the CRISPR/Cas9/dCas9 system is highly versatile, but has imperfect specificity and activity under a wide range of environmental and genotypic conditions [25,28,29], motivating a study of its mechanisms and the development of a model to rationally design its guide RNAs [21]. One major challenge has been binding to off-target DNA sites, resulting in off-target mis-cutting of genomic DNA by Cas9 or gene mis-regulation by dCas9 [28,30,31,32,33]. Several strategies have been shown to reduce Cas9 off-target behavior by manipulating its cleavage activity [33,34,35,36,37,38,39,40]. For example, two guide RNAs expressed together with a partially nuclease-deficient Cas9 nickase have been used to make two single-strand cuts at adjacent locations, increasing the rate of on-target repair by homologous recombination [40]. Further, fusing dCas9 to the FokI nuclease increased the specificity of its nuclease activity to a 20 bp recognition sequence [39]. These strategies address off-target cutting, but not off-target binding and gene regulation. A system-wide understanding of how guide RNAs work together with Cas9/dCas9 to control off- and on-targeting binding would enable the rational design of guide RNAs, and other controllable factors, to improve Cas9/dCas9 specificity and activity. In particular, when engineering dCas9-based genetic circuits, it will be desirable to modulate dCas9's ability to regulate gene expression through the introduction of guide RNA mismatches [8]. However, the quantitative relationship between guide RNA sequence and dCas9's binding affinity is currently unknown.

In this work, we develop a comprehensive, mechanistic model of CRISPR/Cas9 that predicts how experimental conditions and guide RNA sequences (crRNAs) control target site selection and cleavage activity. To initially parameterize this model, we analyze the large amount of structural, biochemical, and next-generation sequencing data that has recently measured several aspects of CRISPR/Cas9's function with different crRNAs under varied experimental conditions [4,29,33,35,37,38,41,42,43,44]. We formulate a single system-wide model that explains how these disparate observations can originate from the same CRISPR/Cas9 mechanism of function. We also present quantitative criteria for designing guide RNA sequences with targeted binding and cleavage activities. By accounting for several important factors beyond the guide RNA sequence, our design rules are a significant improvement over existing, and somewhat contradictory, sequence design rules whose outcomes have also depended on the selected experimental conditions [8,21,33,37,42].

**Fig 1:**
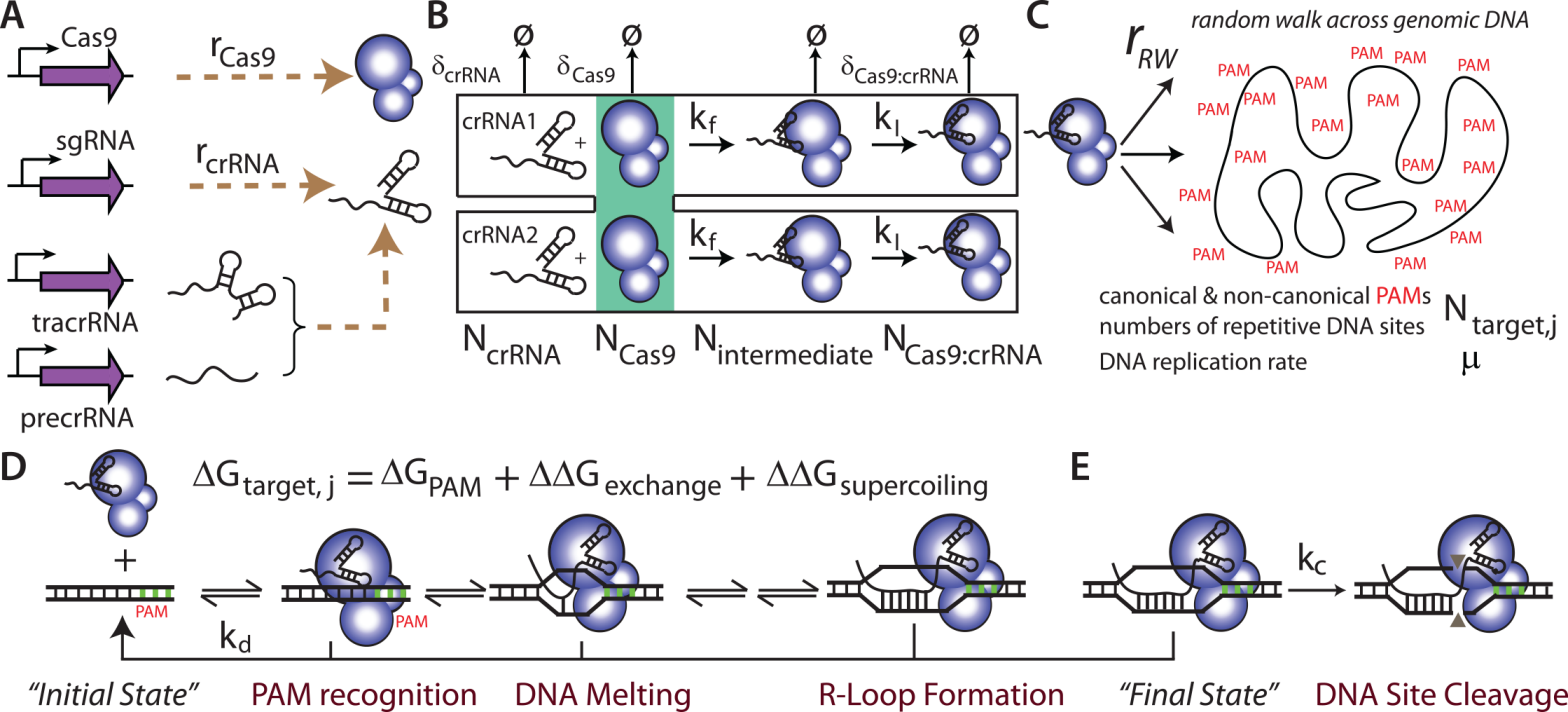
The multi-step mechanism responsible for Cas9-mediated DNA site cleavage. (A) Each crRNA strand is expressed with rate r_crRNA_. The active crRNA is formed by either hybridization of an expressed tracrRNA with an expressed precrRNA or by direct expression of a single guide RNA (sgRNA). The Cas9 endonuclease is expressed with rate r_Cas9_. (B) Cas9 binds to the mature crRNA with a forward kinetic association constant k_f_. After loading the crRNA, the structure of the Cas9:crRNA undergoes an isomerization with forward kinetic constant k_I_ to create an active complex. N_crRNA_, N_Cas9_, N_intermediate_, and N_Cas9:crRNA_ are their numbers of molecules. (C) The resulting active complex performs a 3D random walk with molar flow rate r_RW_. The probability that it binds to a DNA site is determined by the site sequence, including the presence of a protospacer adjacent motif (PAM), the number of same-sequence DNA sites (N_target, j_), and their binding free energy (ΔG_target, j_). (D) The formation of a stable Cas9:crRNA:DNA complex occurs in several steps: Cas9:crRNA recognizes the PAM site, unwinds the DNA duplex, and sequentially replaces DNA:DNA base pairings with RNA:DNA bases pairings in an exchange reaction to form a DNA:RNA:DNA complex, called an R-loop. The DNA target site's binding free energy to Cas9:crRNA (ΔG_target_) sums together its PAM interaction energy (ΔG_PAM_), the energy needed to unwind the supercoiled DNA (ΔΔG_supercoiling_), and the crRNA:DNA exchange energy during R-loop formation (ΔΔG_exchange_). During these steps, the Cas9:crRNA:DNA complex may dissociate with first order kinetic constant k_d_ or it may be cleave the bound DNA site with pseudo first order kinetic constant k_C_. (E) After cleavage, the Cas9:crRNA:DNA complex remains bound to the cleaved DNA, and is considered a no-turnover enzyme. Additional model parameters include the DNA replication rate (μ) and the degradation or dilution rates of Cas9 (δ_Cas9_), crRNA (δ_crRNA_), and Cas9:crRNA complex (δ_Cas9:crRNA_).

**Fig 2:**
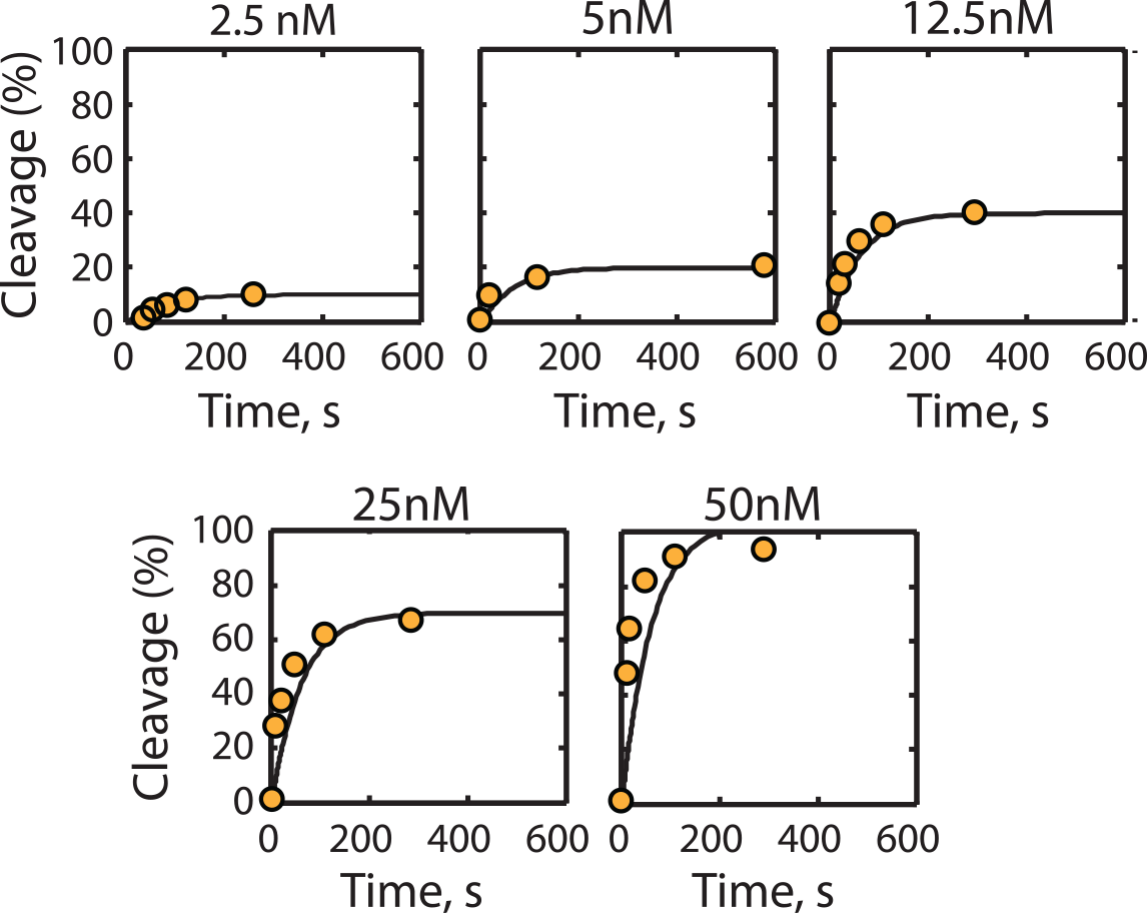
Parameterization of the model using *in vitro* data. Equimolar mixtures of Cas9 and crRNA (concentrations shown) were pre-incubated for 10 minutes, followed by the addition of target DNA and measuring the amount of cleaved DNA. Normalized cleaved DNA measurements (orange circles) using 25 nM negatively supercoiled plasmid DNA are compared to normalized model-calculated amounts of cleaved DNA (lines). Data points represent single measurements from Sternberg et al. [38].

**Fig 3:**
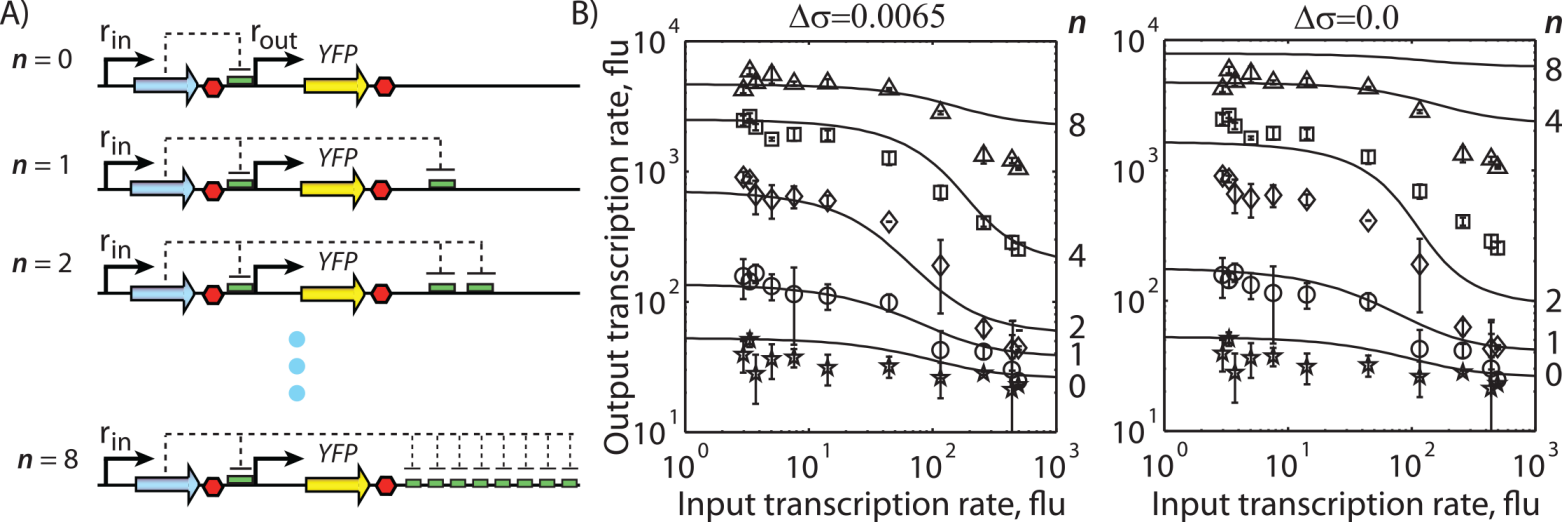
Parameterization of the model using *in vivo* data. (A) The addition of target DNA sites with the same sequence sequesters the Cas9:crRNA complex, and increases the transcription rate of the promoter controlling YFP expression. (B) A comparison between model-calculated transcription rates and measured YFP expression levels when either (stars) 0, (circles) 1, (diamonds) 2, (squares) 4, or (triangles) 8 additional on-target DNA sites were added. The DNA sites’ initial superhelical densities were either (left) increased by 0.0065 per occupied site or (right) kept constant. Data points and bars represent the mean and standard deviation of 2 measurements, performed in this study.

**Fig 4:**
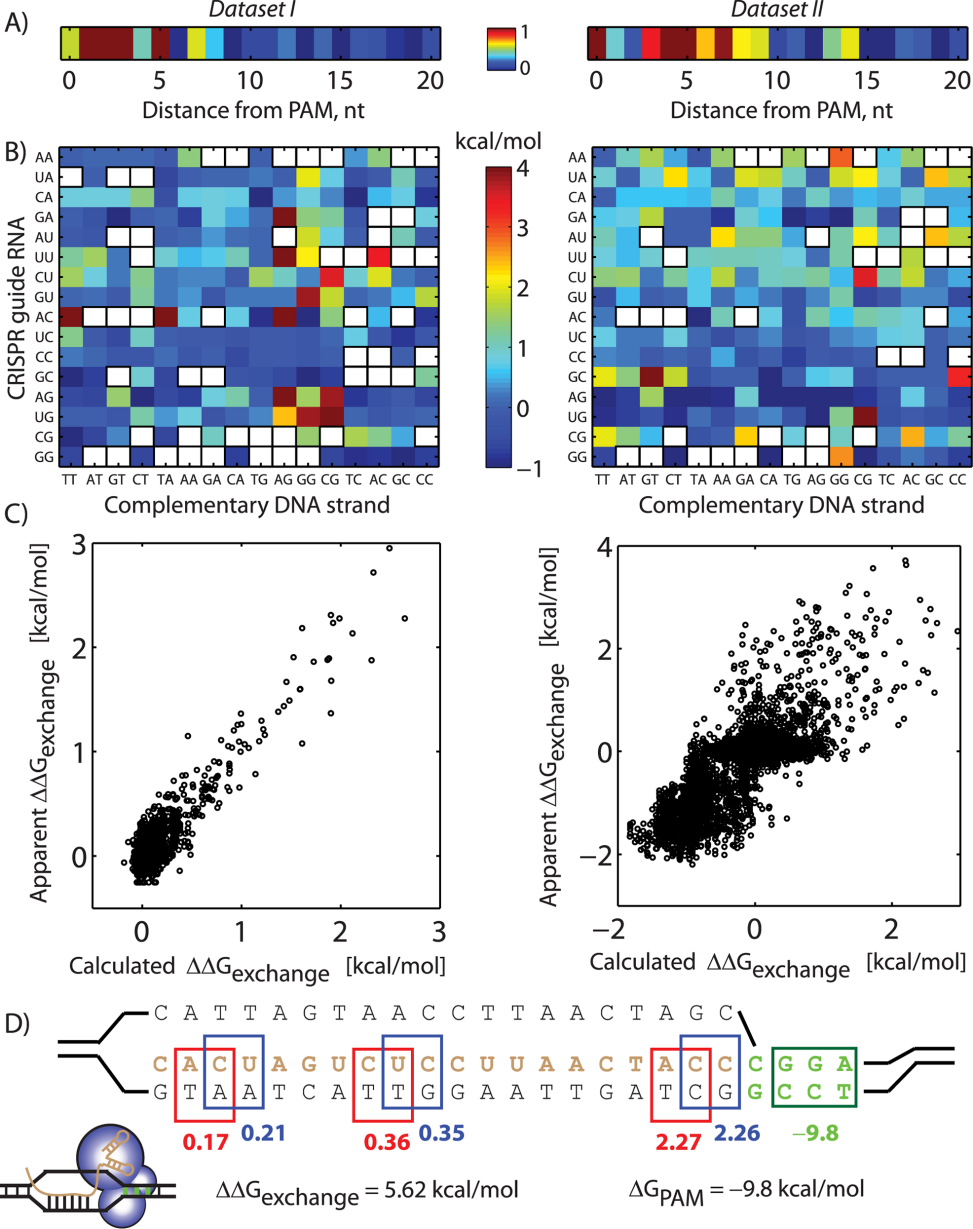
Parameterized free energy models show how mismatched crRNA guide sequences and DNA site sequences affect Cas9 cleavage activity. The (A) 21 position-dependent and (B) 256 sequence-dependent free energy model coefficients were determined using either (left) 3671 *in vitro* Cas9 cleavage measurements from dataset I or the (right) 5979 *in vivo* Cas9 cleavage measurements from dataset II. Coefficients were normalized to their maximum values. White boxes show unidentifiable model parameters, based on the available measurements. (C) Comparisons between apparent and model-calculated ΔΔG_exchange_ across all single measurements. Pearson R^2^ is 0.74 and 0.61, respectively. All points represent single measurements from Pattanayak et. al., Hsu et. al., and Mali et. al [33,37,41]. (D) An example showing how the model is used to calculate ΔΔG_exchange_ and ΔG_PAM_ for a specific guide RNA sequence and DNA site. The energetic contributions of the three mismatches are determined by their (A) position-dependent coefficients and their (B) dinucleotide RNA:DNA identities, using the model parameterized by (left) dataset I. The (green box) PAM sequence determines ΔG_PAM_ using Table 3.

**Fig 5:**
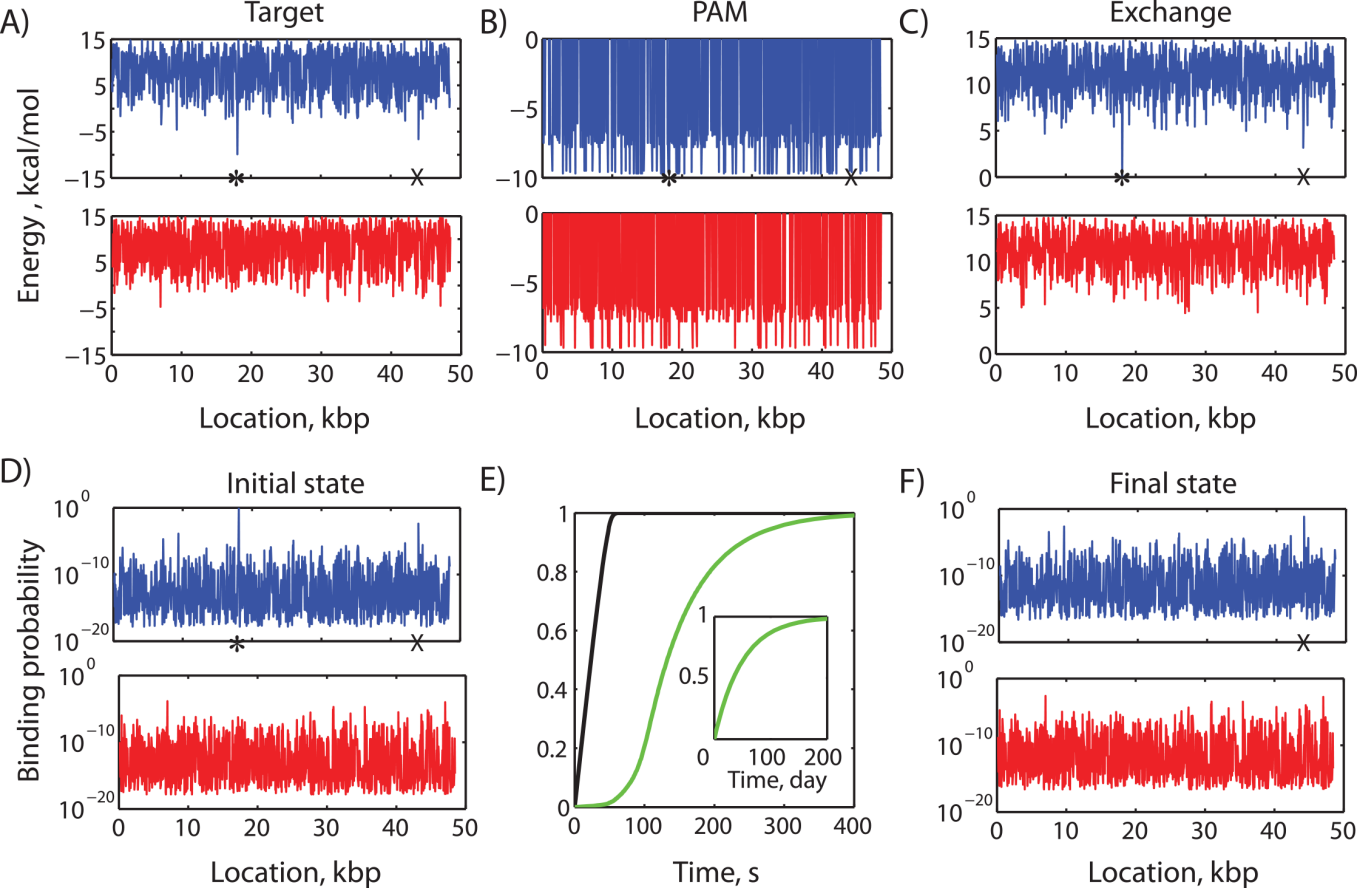
Calculation of dCas9:crRNA_λ2_ binding occupancy across 34,363 PAM sites on a λ-phage genome. (A) Model-calculated target binding free energies (ΔG_target_) are shown across genome position, plotting only one in ten positions for improved visualization. Panels represent either the (top, blue) forward strand or (bottom, red) reverse strand of the λ-phage genome. The target binding free energies are the sum of (B) the free energy change when dCas9 binds to a PAM site (ΔG_PAM_), (C) the free energy change during R-loop formation at PAM-proximal sites, compared to a perfectly complementary sequence (ΔΔG_exchange_), and the free energy change as a result of varying DNA site superhelical density (ΔΔG_supercoiling_). The major on-target site λ2 is denoted by stars. A major off-target site OS1 is denoted by crosses. Here, each mismatch in the crRNA and DNA site sequences contributes up to 0.78 kcal/mol to ΔΔG_exchange_, depending on their distance from the PAM site. The λ-phage genome is assumed to have uniform DNA superhelical density. The model-calculated binding probabilities of (d)Cas9:crRNA_λ2_ to all possible PAM sites are shown at (D) the initial time before any Cas9 activity or (F) after a 10 minute incubation with (d)Cas9:crRNA_λ2_. (E) We show the model-calculated dynamics of (d)Cas9 binding occupancy at the (black line) λ2 DNA site, the (green line) major off-target site OS1, and a (inset) single off-target site with ΔG_target_ = 0 kcal/mol.

**Fig 6:**
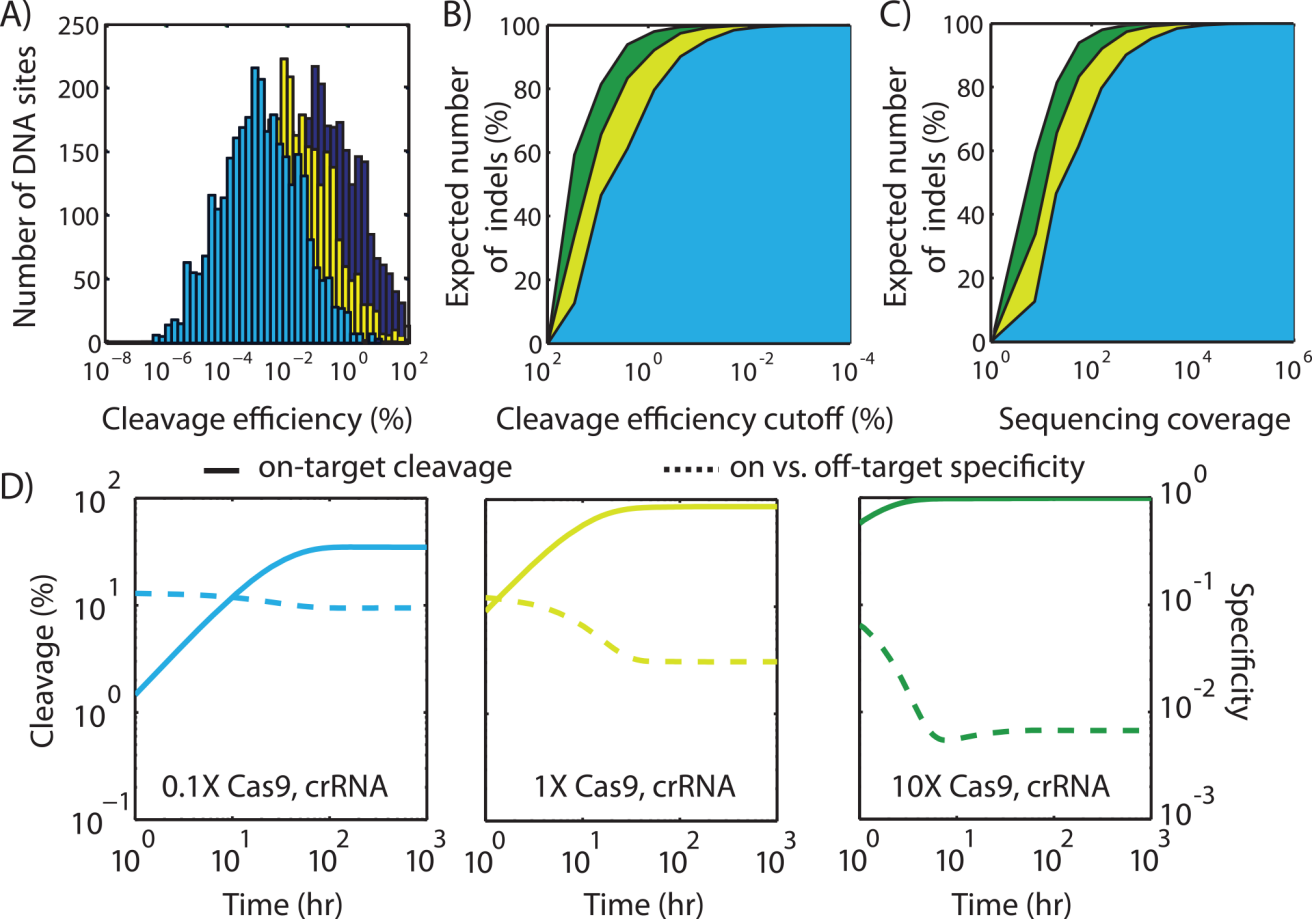
Model predictions for human genome editing. (A) Model-calculated distributions show the numbers of human genome DNA sites that will be cleaved with varying efficiencies when using a LTR-B crRNA with either (yellow) baseline, (blue) 10-fold lower, or (green) 10-fold higher Cas9 and crRNA concentrations. (B) The expected number of off-target indel mutations when counting sites with cleavage efficiencies higher than a cut-off value. (C) The required next-generation sequencing coverage to identify the expected number of off-target indel mutations with 99% certainty. Colors same as in A. (D) The model-calculated dynamics of human genome modification under the same three scenarios, comparing (solid lines) on-target cleavage versus (dashed lines) the ratio between on-target and total off-target cleavage (specificity).

**Fig 7:**
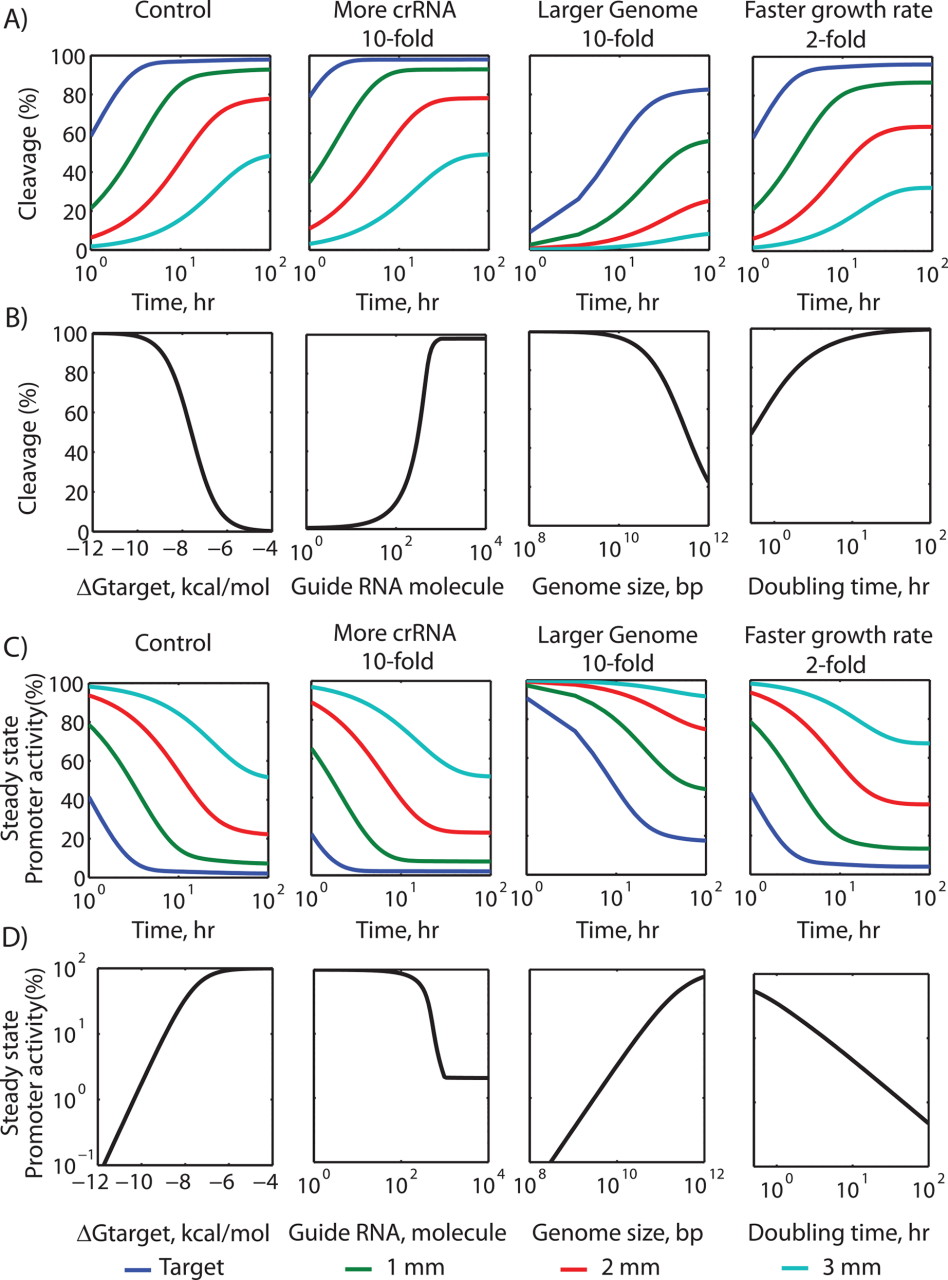
Rational design of genome editing and gene regulation. (A) The dynamics of Cas9-based cleavage at DNA sites with either (blue) zero, (green) one, (red) two, or (cyan) three mismatches, comparing the effects of increasing guide RNA concentration by 10-fold, increasing the genome size by 2-fold, or increasing the cellular growth rate by 2-fold. (B) A sensitivity analysis shows how changing system parameters affect a DNA site’s steady-state cleavage efficiency in growing cells. (C) The dynamics of dCas9-based transcriptional repression (promoter activity) at DNA sites with either (blue) zero, (green) one, (red) two, or (cyan) three mismatches, performing the same comparisons as in A. (D) A sensitivity analysis shows how changing system parameters affect a DNA sites’ steady-state transcriptional repression (promoter activity) in growing cells. mm, mismatch.

To develop this model, we employed statistical thermodynamics and the law of mass action to formulate a five-step mechanism that accounts for concentration-dependent, cell volume-dependent, host genome-dependent, and crRNA-dependent changes to Cas9 complex formation, diffusion, target specificity, and target activity (Fig 1). Kinetic and thermodynamic constants were estimated by analyzing six studies of Cas9/dCas9 function (Table 1). We validated this model using *in vitro* Cas9-dependent cleavage rate data (Fig 2), obtained by Sternberg et al. [38], together with new data collected in this study, measuring *in vivo* dCas9-dependent transcriptional repression in synthetic genetic circuits within bacterial cells (Fig 3). Further, to predict how a guide RNA controls target specificity, we used deep sequencing data [3,33,37,41] to compile a position-dependent, nearest neighbor binding model that accounts for canonical and non-canonical PAM recognition sites, R-loop formation, and mismatches with DNA target sites (Fig 4). We then employ the model to predict the binding occupancies of dCas9 to the lambda phage genome, mirroring a recent experimental study utilizing DNA curtains, to illustrate the differing dynamics between on-target and off-target DNA sites (Fig 5). Finally, we applied the model to predict the frequency and location of off-target cleavage sites in a medically relevant example, where Cas9 was used to remove latent HIV viral DNA segments from a human cell line [45] (Fig 6). Finally, by performing a sensitivity analysis on the model, we show the optimal experimental conditions to maximize on-target (d)Cas9 activity and minimize (d)Cas9 off-target binding (Fig 7).

**Table 1:** A summary of all studies used to estimate the model's parameters.

Parameter	Used in	Reference	Assay	Data used
**ΔG**_**PAM**_	Table 3	Current study	Expression measurements in *E*. *coil*	Multiple on-target sequences
		Jiang et al.[3]	Deep sequencing of cleavage products using *S*. *pneumonia* Cas9.	Cleavage frequencies on randomized PAMs
**k**_**d**_ **, k**_**c**_ **, k**_**f**_ **, k**_**I**_	Fig 2	Sternberg et al. [38]	phospho-imaging of *in vitro* DNA	λ2 target on plasmid & synthesized DNA
		Sternberg et al. [38]	Fluorescently labeled dCas9 on *in vitro* DNA curtains	—
		Szczelkun et al.[44]	Magnetic tweezers measuring DNA supercoiling *in vitro*	Single molecule and bulk assays
**ΔΔG**_**supercoiling**_	Fig 2	Depew and Wang[48]	Electrophoresis of plasmids with topoisomer distribution	—
**Δσ**	Fig 3	Current study	Expression measurements in *E*. *coli*	Multiple on-target sequences
**ΔΔG**_**exchange**_ **(dataset I)**	Fig 4	Pattanayak et al.[33]	Deep sequencing of *in vitro* Cas9 reaction products using sgRNA guides	CLTA1, CLTA2, CLTA3
**ΔΔG**_**exchange**_ **(dataset II)**	Fig 4	Hsu et al.[37]	Deep sequencing of Cas9 products in HEK 293FT cell line using sgRNA guides	Emx1.1, Emx1.2, Emx1.3, Emx1.6, Emx1.10,Emx1.11, Emx1.12,Emx1.13, Emx1.14, mx1.15,Emx1.16,Emx1.17, Emx1.18,Emx1.19,Emx1.20
		Mali et al.[41]	Deep sequencing of Cas9 products in HEK 293T cell line using gRNA guides	gRNA1
		Pattanayak et al.[33]	Deep sequencing of *in vitro* Cas9 reaction products using sgRNA guides	CLTA1, CLTA2, CLTA3, CLTA4

## Results

### A Mechanistic Model of CRISPR/Cas9

The activity of Cas9-mediated cleavage is dictated by a 5-step mechanism that includes the expression of Cas9 and crRNA, the formation of active Cas9:crRNA complex, a random intracellular walk to search for DNA target sites, the formation of a Cas9:crRNA:DNA complex (an R-loop) at DNA sites, and finally DNA site cleavage (Fig 1). We developed a dynamical mechanistic model that incorporates all known biomolecular interactions and processes that control the rates of these steps (Materials and Methods). The mechanistic model accounts for how several factors control all the DNA sites' cleavage rates, including changing Cas9 and crRNA expression levels, different crRNA protospacer (guide) sequences, different DNA site sequences, both canonical and non-canonical PAM recognition DNA site sequences, and the effects of DNA site supercoiling. The model also explicitly accounts for the host's specifications, including its genome sequence, genome length, cell size, and growth rate. Moreover, the model allows for the expression of multiple crRNA guide strands, and it will determine how the competitive binding of crRNAs to Cas9 will also affect the DNA sites' cleavage rates. When expressing Cas9, the model calculates the numbers of all free, bound, and cleaved DNA sites that contain a canonical or non-canonical PAM site, encoded within the host genome or on plasmids. When expressing nuclease-deficient dCas9, the model calculates the occupancy of stably bound dCas9:crRNA complexes to all DNA sites. Overall, the formulated model contained eight unknown parameters quantifying the binding interactions between Cas9 and crRNA as well as the effects of DNA site supercoiling on Cas9 binding affinity. In addition, the model also utilized a multi-parameter free energy model quantifying crRNA-DNA site interactions.

### Model Parameterization Using *In Vitro* Measurements

We first utilized the *in vitro* measurements obtained by Sternberg et. al. to determine the kinetic parameter values that quantify Cas9:crRNA complex formation, pre-cleavage dissociation, and Cas9-dependent cleavage [38]. In this study, the binding locations and cleavage rates of Cas9 using a plasmid DNA substrate were measured to characterize the multi-step process by which Cas9 finds DNA targets, initiates R-loop formation, and cleaves DNA sites. Here, we utilized the authors' dynamic measurements of DNA site cleavage at different concentrations of Cas9 and crRNA, using either an on-target site on plasmid DNA (Fig 2B in [38]) or an on-target site on a double-stranded DNA fragment (Extended Data Fig 5 in [38]). We also analyzed Cas9’s protein structure and its motility to estimate that Cas9's characteristic length is λ_Cas9_≈150°A [43,46] and its diffusivity in a cytoplasmic-like buffer is 45 μm^2^/s [47]. Therefore, we determined that Cas9 performs an isotropic random walk with a diffusive specific flow rate of 4.05 x10^-10^ 1/sec. In the presence of 25 nM plasmid DNA, these calculations indicate that a Cas9:crRNA complex collides with a DNA site 61 times per second.

We then determined the kinetic parameter values controlling Cas9:crRNA association (k_f_), isomerization (k_I_), pre-cleavage dissociation (k_d_), and cleavage activity (k_C_) by calculating the rate of cleavage (r_C_) across a range of Cas9 and crRNA concentrations, mirroring the experimental conditions, and comparing to experimental cleavage measurements (56 experiments; R^2^ = 0.97; S1 Fig) using 25 nM plasmid DNA [38]. The model solution was evaluated for an initial 10 minute time period, followed by *in silico* addition of the DNA substrate and an additional 30 minute time period. The best-fit kinetic parameter values were then determined through optimization to minimize the relative error between calculated and measured cleavage rates (Materials and Methods). Based on our analysis (S2 Fig), we could uniquely parameterize k_f_, k_I_, and the ratio k_c_/k_d_ (Table 2). Surprisingly, the rate of cleavage was found to be less than the rate of pre-cleavage dissociation (k_c_/k_d_ << 1), suggesting that (d)Cas9 must engage in multiple aborted rounds of binding and R-loop formation before successfully cleaving the DNA site. Using the best-fit parameter values, the model was able to accurately capture the experimentally observed time-dependent cleavage rates while varying the Cas9 and crRNA concentrations (Fig 2). The best-fit parameter values are reported in Table 2.

**Table 2:** Parameter values used in this study.

Parameters	Value	Units
**σ**_**F**_	-0.1	Turns / 10 bp
**σ**_**NS**_	-0.06	Turns / 10 bp
**k**_**c**_ **/ k**_**d**_	0.0016	unitless
**k** ^*^_d_	5	1/sec
**Δσ**	0.0065	turns / 10 bp
**k**_**I**_	1	1/sec
**k**_**f**_	0.08	1/sec
**ΔG**_**PAM,ref (CGGTA)**_	-9.9	kcal/mol
**ΔΔG**_**single-mismatch**_	0.78	kcal/mol

As expected, when the Cas9 concentration is limiting, the calculated amount of cleaved DNA is almost equal to the Cas9 concentration because Cas9 does not turn-over. However, when non-supercoiled, short (55 bp) DNA fragments were used as template, Sternberg et. al. found that Cas9’s total cleavage activity dropped by 5-fold even though the apparent cleavage rate of DNA increased (S3A Fig). The authors hypothesized that the reduced cleavage activity originated from a batch of partially active Cas9 enzyme. To test this possibility, we first reduced the concentration of Cas9 *in silico* to 20% of the reported concentration. The model reproduced the measured amount of cleaved DNA after the 10 minute incubation period, however, the model-calculated rise to steady-state was slower than the experimentally observed rise (S3A Fig). Instead, if we also accounted for the much smaller number of DNA sites and the lack of negative supercoiling of the short DNA fragments, then the model correctly explains the experimentally observed fast rise time (S3B and S3C Fig). Specifically, there were 5482 total possible DNA sites (N) when plasmid DNA template (2741 bp) was used in the *in vitro* measurements, compared to only 110 possible DNA sites when short DNA fragments were used (55 bp), resulting in about 50-fold higher rise time. The difference in DNA site supercoiling partly counteracted this much higher model-calculated rise time by requiring an additional 0.43 kcal/mol energy for the Cas9:crRNA complex to successfully form an R-loop, lowering the model-calculated rise time to about 25-fold higher than when using the plasmid DNA as template, which is close to the experimental measurement.

### Quantifying the Effects of Supercoiling on Adjacent DNA sites

When using dCas9 to implement genetic forms of computing, we anticipated the need to introduce several adjacent crRNA binding sites to differentially regulate gene expression. However, according to the biophysics of R-loop formation, it was possible that the binding of a (d)Cas9:crRNA complex to one target DNA site could actually lower the affinity of (d)Cas9:crRNA complexes to adjacent DNA sites. Specifically, when a dCas9:crRNA complex binds to a DNA site, the creation of an R-loop will negatively supercoil the site’s DNA, for example, by untwisting it. Because DNA’s linking number is conserved, the negative supercoiling of one DNA site will increase the positive supercoiling of adjacent DNA sites. According to model Eq 13, a higher superhelical density will make it less likely for another dCas9:crRNA complex to bind to adjacent DNA sites by requiring a higher free energy input to stably form an R-loop [48].

To investigate this effect, we constructed a three plasmid system that expresses dCas9 using a constitutive promoter, a single crRNA using an IPTG-inducible P_TAC_ promoter, and a YFP reporter protein using a constitutive promoter containing a fully complementary (on-target) crRNA binding site (Fig 3A). Using dCas9:crRNA as a transcriptional repressor, we measured steady-state YFP expression levels as the transcription rate of the crRNA was steadily increased via IPTG induction. We then introduced either one, two, four, or eight additional on-target crRNA binding sites at a distal location on the high-copy reporter plasmid, upstream of the YFP promoter, separated by a transcriptional terminator, and performed the same YFP fluorescence measurements. These auxiliary on-target crRNA binding sites were separated by 60 to 80 bp of non-repetitive DNA. The presence of the many additional crRNA binding sites in a non-regulatory location had the expected effect of sequestering dCas9:crRNA, resulting in lower amounts of transcriptional repression at YFP’s promoter and higher YFP expression levels (Fig 3B).

In light of this data, we consider two distinct hypotheses relating the number of artificially added crRNA binding sites to the apparent increase in YFP expression level. First, if dCas9-mediated R-loop formation has no effect on the superhelical density of surrounding crRNA binding sites, then we should expect that adding more crRNA binding sites will proportionally sequester more dCas9:CrRNA, resulting in greater YFP expression levels as more crRNA binding sites are added. Second, if dCas9-mediated R-loop formation does increase the supercoiling of adjacent DNA sites, then we should expect that many additional crRNA binding sites will not proportionally sequester more dCas9:crRNA, resulting in a sub-linear increase in YFP expression as more sites are added. To quantify the extent that R-loop formation increases the superhelical density of surrounding crRNA binding sites, we added a single parameter to our model (Δσ). When *n* copies of dCas9:crRNA are bound to nearby DNA sites, the initial superhelical density of the remaining nearby DNA sites is increased by *n*Δσ, which increases the sites’ ΔG_supercoiling_ according to Eq 13, and lowers the probability that they will be bound by additional dCas9:crRNA. If Δσ is zero, model calculations show that adding 8 crRNA binding sites to the plasmid will yield greater amounts of dCas9:crRNA sequestration, resulting in 300-fold more YFP expression (Fig 3B, right). However, if Δσ is positive, adding more crRNA binding sites will yield diminishing amounts of dCas9:crRNA sequestration and sub-linear increases in YFP expression (Fig 3B, left).

Using this data-set to evaluate these two hypotheses, we found that adding 2, 4, or 8 additional crRNA binding sites increased dCas9:crRNA sequestration and YFP expression, but with lower-than-proportional amounts, suggesting that there is indeed a anti-cooperative mechanism affecting site occupancies (Fig 3B). We found that a moderate site-to-site superhelical density penalty (Δσ = 0.0065) was sufficient to explain how adding more crRNA binding sites sublinearly increased dCas9:crRNA sequestration and YFP expression level (Fig 3B, left) with a high degree of confidence (R^2^ = 0.97, p < 10^−8^; S4 Fig). The apparent site-to-site changes in superhelical density appear to be additive; for dCas9 to stably bind to the 8 binding site array, it would be necessary to untwist over 160 bp of the 900 bp region, equivalent to about 6 kcal/mol of free energy input, which would greatly destabilize R-loop formation and lower dCas9:crRNA occupancy. To compare, a model that ignores changes in superhelical density, and its effect on dCas9:crRNA occupancy, was not able to explain the measurements (Fig 3B, right). Additionally, according to this data-set, it appears that crRNA concentration, and not dCas9 concentration, was limiting the total amount of dCas9:crRNA that could bind these additional crRNA bind sites or the promoter to repress YFP expression, discounting an alternative hypothesis.

### Model Parameterization for Canonical and Non-Canonical PAM Sites

Cas9 requires the presence of a protospacer adjacent motif (PAM) sequence to bind to a DNA site, form an R-loop, and cleave DNA. While the consensus PAM sequence for the Cas9 from *S*. *pyogene*s is NGG, it was previously observed that R-loop formation could take place at non-canonical PAM sites, resulting in a considerable amount of off-target activity [3,35,42]. To quantify Cas9’s binding free energy to DNA sites that use either canonical and non-canonical PAM sites, we utilized data from a recent study that measured Cas9’s cleavage activity when bound to DNA sites with identical PAM-proximal sequences, but randomized PAM sequences, using a homolog of Cas9 from *S*. *pneumonia* [3]. We compared cleavage activities to a reference PAM site, which we defined by the four nucleotide sequence 5’-CGGT-3’, with a corresponding reference free energy (ΔG_PAM,ref_ = -9.9 kcal/mol). This reference free energy was consistent with our *in vivo* measurements shown in Fig 3. Importantly, we found that the first nucleotide (N in NGG) did not significantly contribute to Cas9’s cleavage activity, but that the fourth nucleotide did significantly alter cleavage activity. We then employed model Eqs 6 and 15 to calculate the change in ΔG_target_, and therefore the change in ΔG_PAM_, corresponding to each four nucleotide PAM sequence. As only the PAM sequences vary, the free energies ΔG_exchange_ and ΔG_supercoiling_ were not expected to change significantly. To eliminate background noise, we excluded any PAM sequence that resulted in less than 1% cleavage. Further, we found that averaging cleavage activities over the first nucleotide position of each PAM sequence resulted in apparent free energies with a low coefficient of variation of 9%. Overall, we quantified the apparent ΔG_PAM_ free energies of 26 PAM sequences and found that they vary by 4 kcal/mol (Table 3), which is equivalent to about 700-fold change in instantaneous cleavage activity (all other factors being equal).

**Table 3:** Apparent Cas9 binding energies to canonical and non-canonical PAM sites (kcal/mol). The energies are average values of all combinations in the first and fifth positions. (blue) The canonical PAM sites (NGGN) are bolded. N.B: no statistically significant binding. nt: nucleotide.

2nd nt position	A	AAA	N.B.	ATA	N.B.	ACA	N.B.	AGA	-7.5	A	4^th^ nt position
		AAT	-6.6	ATT	-6.8	ACT	N.B.	AGT	-7.7	T	
		AAC	N.B.	ATC	-6	ACC	N.B.	AGC	-7.7	C	
		AAG	-6.6	ATG	N.B.	ACG	N.B.	AGG	-7.7	G	
	T	TAA	N.B.	TTA	N.B.	TCA	N.B.	TGA	N.B.	A	
		TAT	-6.8	TTT	-7.2	TCT	N.B.	TGT	N.B.	T	
		TAC	N.B.	TTC	N.B.	TCC	N.B.	TGC	N.B.	C	
		TAG	-6.8	TTG	-6.4	TCG	N.B.	TGG	-7.4	G	
	C	CAA	N.B.	CTA	N.B.	CCA	N.B.	CGA	N.B.	A	
		CAT	N.B.	CTT	N.B.	CCT	N.B.	CGT	N.B.	T	
		CAC	N.B.	CTC	N.B.	CCC	N.B.	CGC	N.B.	C	
		CAG	N.B.	CTG	N.B.	CCG	N.B.	CGG	-7.7	G	
	G	GAA	-6.8	GTA	-7	GCA	N.B.	**GGA**	-9.4	A	
		GAT	-6.9	GTT	-7.5	GCT	-6.7	**GGT**	-9.6	T	
		GAC	-6.8	GTC	N.B.	GCC	N.B.	**GGC**	-9.7	C	
		GAG	-6.9	GTG	-7.3	GCG	-6.5	**GGG**	-9.5	G	
		A		T		C		G			
	3rd nucleotide position										

As expected, the canonical PAM site NGGN binds with the highest affinity to Cas9 with ΔG_PAM_ energies exceeding -9 kcal/mol. However, there are several non-canonical PAM sites with sufficiently high affinities to contribute to off-target cleavage activity, including NAGN and NGWN. Further, the presence of a gap between a fully complementary protospacer and a PAM site does not fully ablate Cas9’s binding affinity; a single nucleotide gap (NNGG) penalized binding by 2.2 kcal/mol, while a single nucleotide bulge (GGNN) had a larger effect (a 3 kcal/mol penalty). Recent studies have demonstrated that Cas9 can bind well to several non-canonical PAM site such as NAG, NGA, NAA, NTG, NGC, NCG, and NGT, though the extent of its promiscuity does depend on the Cas9 species origin [49,50]. Using the ΔG_PAM_ free energies in Table 3 and an estimate of the DNA site’s superhelical density, the model can now calculate the binding free energy (ΔG_target_) of Cas9:crRNA when the crRNA’s guide sequence perfectly matches the DNA site’s sequence. To quantify the effects of mismatches, we next developed a free energy model (ΔΔG_exchange_) that accounts for changes in the crRNA's guide sequence.

### Modeling the Effects of crRNA:DNA Site Mismatches

A mismatch between the crRNA guide sequence and a DNA site destabilizes the formation of the Cas9:crRNA:DNA R-loop and increases the likelihood that the Cas9:crRNA complex dissociates prior to cleaving the DNA site [35,38,44]. In our model, we quantify the thermodynamics of the R-loop strand displacement process, comparing the free energy of the initial double-stranded DNA state to the free energy of the Cas9:crRNA:RNA R-loop, resulting in a free energy change (ΔΔG_exchange_). ΔΔG_exchange_ will change whenever a mismatch is introduced, though the magnitude of the change will depend on both the position of the mismatch and the surrounding sequence composition. As the last step in developing our model, we utilized three next-generation sequencing datasets (Table 1) to parameterize position- and sequence-dependent free energy models quantifying the Cas9:crRNA:DNA interactions during R-loop formation. Three types of free energy models were created and compared to investigate whether Cas9 plays a role in mediating these interactions, and whether these interactions varied across different host genomes.

In the Pattanayak et al., the on-target and off-target cleavage activities from four sgRNAs were measured via deep sequencing across a degenerate library of DNA sites within an *in vitro* reaction [33]. In Hsu et. al. and Mali et. al., respectively, the amounts and locations of Cas9-based cleavage and dCas9-based transcriptional activation were recorded *in vivo* via deep sequencing [33,37,41]. We categorized these measurements into two data-sets, dataset I and II (Table 1). To analyze these data-sets, we first identified all DNA sites that utilized a canonical PAM sequence similar to the PAM sequence adjacent to the targeted sequences and yielded greater than 50 read counts, finding 3671 sites in data-set I and 5979 sites in data-set II. Further, the superhelical densities of DNA sites are the same within the *in vitro* data-set, and largely similar across the *E*. *coli* genome, enabling us to disregard changes in ΔΔG_supercoiling_ for this analysis. We then compared sequencing read counts between Cas9 cleavage at the perfectly complementary (on-target) site and all off-target sites, obtaining a direct relationship between changes in sequencing read count and changes in ΔΔG_exchange_, according to our model Eqs 7 and 8. When analyzing dCas9-based transcriptional activation measurements, we assumed that the dCas9 binding probability was proportional to the transcription rate of the target promoters. For each sequence, this rate was also proportional to the ratio of the background-subtracted read counts from the samples and the background-subtracted read counts from the positive controls. We then utilized Eqs 7 and 8 to convert the normalized RNA-Seq read counts into changes in ΔΔG_exchange_ [51]. By excluding alternative PAM sites, we were able to more precisely quantify the energetic effects of introducing mismatches into DNA site sequences.

Comparing the Pattanayak et. al. and Hsu et. al. datasets, the overall average energetic penalty for a single mismatch was 0.14 and 0.78 kcal/mol, equivalent to a 1.26-fold and 3.7-fold drop in Cas9 activity, respectively, which suggests that the differences between *in vivo* and *in vitro* measurements and characterization protocol had an influence on off-target cleavage activities. However, some single mismatches were found to penalize ΔG_exchange_ by 4 kcal/mol, equivalent to a 785-fold drop in Cas9 activity. Therefore, we next formulated position-dependent and sequence-dependent models to quantify how the introduction of mismatches in either the crRNA guide sequence or DNA site sequence affected Cas9 activity.

In the first free energy model, we employed Eq 9 to calculate ΔΔG_exchange_, which quantifies the thermodynamic stability of the RNA-DNA and DNA-DNA complexes responsible for R-loop formation, together with 21 unknown position-dependent coefficients. While the free energies of DNA-DNA and DNA-RNA complementary duplexes have been measured [52] [53], there has been limited measurements of DNA-RNA mismatch free energies. Using a dinucleotide nearest-neighbor model, there are 240 types of RNA-DNA mismatches; however, the free energies of only about 72 of them have been experimentally measured [54,55,56,57,58]. After incorporating the known complementary and mismatch DNA-DNA and RNA-DNA free energies into Eq 9, and utilizing either dataset I or dataset II to parameterize the position-dependent coefficients, the resulting model was not able to predict Cas9 binding or cleavage activity (R^2^ = 0.32 and 0.07 for dataset I and dataset II, respectively; S4 Fig). Consequently, we anticipate that additional measurements of RNA-DNA mismatch free energies and kinetic modeling will improve the development of accurate first principles models of R-loop formation.

We then developed an alternative free energy model (Eq 10) that does not rely on previous thermodynamic measurements of nucleic acid interactions, but instead uses measured Cas9 activities at thousands of DNA sites to determine unknown model parameters. The free energy model accounts for all possible guide RNA guide sequences and DNA site sequences, employing a dinucleotide nearest-neighbor model (256 unknown coefficients) together with 21 position-dependent coefficients. We determined the unknown parameters using either dataset I (3671 measurements) or dataset II (5979 measurements), utilizing nonlinear least-squares to minimize the error between the apparent and calculated ΔΔG_exchange_ free energies (Materials and Methods). This parameterization determined values for 86% and 80% of the unknown parameters, using dataset I and II, respectively. In particular, these datasets lacked DNA sites with two consecutive mismatches, resulting in several unidentified parameters. The resulting free energy models for ΔΔG_exchange_ were qualitatively consistent with anecdotal observations; for example, the first eight position-dependent coefficients have the highest values, accounting for about 67% (dataset II) to 81% (dataset I) of ΔΔG_exchange_ variation, quantifying the impact of PAM-proximal mismatches on Cas9 activity (Fig 4A). As a comparison, in a recent *in vivo* study [29], 87% of sequences with high binding affinities to a Cas9:crRNA complex have at most 1 mismatch within the first 8 nucleotides (Fig 4B). The apparent mismatch free energies also varied up to 5 kcal/mol, suggesting that mismatch sequence composition is an additional factor that affects Cas9 activity. However, the energetic penalties of specific mismatched RNA:DNA sequences were not necessarily the same across the two models. When parameterized with *in vitro* Cas9 cleavage measurements (dataset I), the most energetically unfavorable mismatches were found at dAG, dGG, and dCG dinucleotides that were positioned over rAC, rAG/rGA, or rGT/rTG dinucleotides. In contrast, when parameterized with *in vivo* Cas9 activity measurements (dataset II), the mismatch free energy penalties were more evenly distributed, potentially due to confounding interactions arising from the DNA sites' chromatin states.

Overall, the empirically parameterized free energy models were able to sufficiently account for the sequence- and position-dependent effects on Cas9 activity across the thousands of DNA sites (R^2^ = 0.74 and 0.61 for datasets I and II, respectively; Fig 4C). However, the maximum uncertainty in a free energy parameter was 2 kcal/mol, indicating that there is significant opportunity for improving both the breadth and precision of Cas9 activity measurements with the objective of developing more accurate free energy models.

### Predicting dCas9 Binding Occupancy across the Lambda Bacteriophage Genome

Next, we applied the parameterized mechanistic model to calculate dCas9 binding occupancies across the lambda bacteriophage genome when using a crRNA guide sequence that targets a specific genomic location, designated λ2. Our calculations mirror recently conducted experiments that monitored the dynamics of fluorescently labeled dCas9:crRNA_λ2_ as it interacted with an array of λ-phage genomic DNA within a flow chamber, called a DNA curtain [38]. Using these calculations, we examine how the sequence composition and PAM density of a genome affects the partitioning of dCas9 and its binding dynamics.

Overall, the λ-phage genome contains 3179 and 2497 canonical PAM sites on its forward and reverse strands, respectively, together with 17933 and 16445 non-canonical PAM sites with a density of about one PAM site per 2.4 bp. To calculate the dCas9 binding free energies at all PAM sites, we identified their corresponding ΔG_PAM_ binding free energies (Table 3) and used both the λ2 guide and DNA site sequences to calculate the free energy change during R-loop formation (ΔΔG_exchange_). Here, we utilized the previously parameterized distance-dependent coefficients (Fig 4) and a DNA:RNA mismatch penalty of 0.78 kcal/mol, which was the overall average energetic penalty observed in the Hsu et. al. data-set. We also assumed that all λ-phage genomic sites are equally supercoiled (ΔΔG_supercoiling_ = 0). Model parameters are listed in S1 Table.

We found that the binding free energies of dCas9:crRNA_λ2_ varied by 25 kcal/mol across the 40054 PAM sites, and only 3880 of them had negative dCas9:crRNA_λ2_ binding free energies (ΔG_target_ < 0) (Fig 5A). Most PAM-proximal DNA sites had large numbers of mismatches with the crRNA_λ2_ guide sequence, causing ΔΔG_exchange_ to be more positive than ΔG_PAM_ (Fig 5B and 5C). In particular, there were only 25 DNA sites that had highly negative binding free energies (ΔG_target_ < -6 kcal/mol). As expected, the λ2 DNA site formed a perfect DNA:RNA duplex with crRNA_λ2_, resulting in a zero model-calculated ΔΔG_exchange_ penalty and a ΔG_target_ of -9.9 kcal/mol. However, a second off-target DNA site, designated OS1, had a canonical PAM (GGGA, ΔG_PAM_ = -9.4 kcal/mol), only two mismatches within the 8 most PAM-proximal nucleotides, and an additional six mismatches in the remaining 12 nucleotides, yielding a ΔG_target_ of -6.3 kcal/mol. Interestingly, fluorescently labeled dCas9 was observed to transiently bind to OS1’s position in the λ-genome [38]. By enumerating and calculating the dCas9 binding free energies for all PAM sites, we can then calculate the system’s overall partition function to determine their binding occupancies under several scenarios.

The canonical partition function quantifies the amount of dCas9:crRNA that will be sequestered under equilibrium conditions. It is also used in Eq 7 to determine the instantaneous binding probabilities to all DNA sites. When using dCas9:crRNA_λ2_, a fully accessible λ-genome has an overall partition function value of 162.6. The λ2 DNA site contributes the largest amount (151.04), indicating that it has the largest probability of being bound first. The off-target OS1 site contributes only 0.37 to the partition function summation, and therefore has a 408-fold lower probability of being bound first, compared to λ2. However, the additional 3879 off-target sites provide a significant contribution to the partition function summation, which will affect the binding occupancies at all PAM sites; sites with canonical PAMs contribute 7.83, while those with non-canonical PAMs contribute 3.36. As a result, it is 30-fold more likely that dCas9:crRNA_λ2_ will initially bind to one of these minor off-target sites, compared to the major off-target site OS1. Rather than searching only for PAMs with the most complementary DNA sites, it becomes important to enumerate all possible PAM sites to correctly determine their partition function contributions.

We next applied the mechanistic model to determine how dCas9 binding occupancies to the λ-phage DNA curtains will change over time. Here, mirroring the experimental system, we assume constant dCas9 and crRNA_λ2_ concentrations of 10 nM and 100 nM, respectively, along with a system volume of 100 μL. While our initial partition function calculations assumed that all λ-genome DNA sites are equally accessible, as dCas9:crRNA diffuses and binds to its DNA targets, it will irreversibly sequester DNA sites and eliminate their contributions to the partition function. By substituting our partition function calculations into the model’s system of differential equations (Materials and Methods), using parameters listed in Table 2, we calculated how the numbers of accessible DNA sites change over time, which then alters the binding probabilities of the remaining DNA sites (Fig 5D and 5F).

As expected, the λ2 DNA site binds fastest to dCas9:crRNA_λ2_ and is predicted to be fully bound within a minute (Fig 5E). During that time, the average binding occupancies of the other individual DNA sites do not appreciably increase. However, once the λ2 DNA site has been sequestered, the system’s overall partition function decreases from 162.6 to 11.56, which increases the off-target binding rate of dCas9:crRNA_λ2_ by 14-fold. As a result, the OS1 major off-target DNA site becomes fully bound within the next 6 minutes. Then, once the OS1 off-target site has been sequestered, the remaining off-target DNA sites become the only possible locations where dCas9:crRNA_λ2_ can bind. DNA sites with ΔG_target_ energies of -4, -2, and 0 kcal/mol will become fully bound after 6 hours, 7 days, and 200 days, respectively. These calculations assume that DNA sites remain indefinitely sequestered after dCas9 irreversibly binds, which is correct for this scenario. However, in growing cells, unbound DNA sites are continuously replenished through DNA replication according to the cell’s growth rate.

### Predicting the Frequency and Locations of Off-Target Cleavage during Human Genome Editing

For Cas9-based genome editing to become reliably used for therapeutic applications, the factors that determine the frequency and location of its off-target cleavage activity must be better understood. Here, we applied the mechanistic model to calculate the distribution of all possible off-target cleavage sites during Cas9-based human genome editing and the necessary next-generation sequencing coverage to detect the resulting indel mutations with high certainty. As a clinically relevant example, our calculations mirrored a recent study that applied Cas9-based genome editing to excise integrated copies of the HIV provirus from infected human U1 cell lines by cleaving genomic DNA at flanking LTR sites [45].

Using the parameterized model, we first calculated the binding free energies between Cas9:crRNA_LTR-B_, using a guide RNA that complements the LTR-B recognition sequence, and all off-target DNA sites within the reference human genome, finding that there are 3105 DNA sites with negative binding free energies (ΔG_target_ < 0) [59]. We repeated these calculations on a single copy of the HIV provirus, including the on-target LTR-B site. We solved the model’s system of differential equations describing time-dependent cleavage at both on- and off-targets sites to determine their cleavage efficiencies after a 1000 hour time period, using the kinetic parameters in Table 2, a genome length (N) of 6.4x10^9^ bp (forward and reverse strands), a 8-fold higher system volume, a cell doubling time of 20 hours, and 100 nM initial concentrations for both Cas9 and crRNA_LTR-B_ (S2 Table).

The resulting cleavage efficiencies varied considerably across eight orders of magnitude; the on-target LTR-B site reached 100% cleavage, while the off-target sites had cleavage efficiencies ranging from 1 in 10,000,000 (10^−5^%) to 85% (Fig 6A). The majority of off-target sites have extremely low cleavage efficiencies (less than 1%), creating a mixture of cells with high genomic heterogeneity. Consequently, if we assume that cleavage events become indel mutations, then identifying their locations across an entire genome will require a highly sensitive indel-specific assay or next-generation sequencing with high coverage. For example, to detect 50% of all expected indel locations across a genome, an assay must be capable of positively identifying the presence of an indel at a single location even if its frequency is only 1 in 5 (20%) within the genome mixture (Fig 6B). The assay must be 20-fold more sensitive (1 in 100) to detect 90% of all indel locations. When using next-generation sequencing for detection under a best-case scenario, at least 13X coverage will be needed to identify 50% of all expected indels and at least 200X coverage to identify 90% of all indels (Fig 6C).

Using the model, we then determined how the DNA sites’ cleavage distributions were affected when lowering the Cas9 and sgRNA concentrations by 10-fold (Fig 6A, blue). The distribution shifted leftward and the off-target DNA sites’ average cleavage efficiency decreased from 1% to 0.1%. While decreasing the frequency of indels is the prime objective, an even more sensitive assay will be needed to confirm their absence. According to model calculations, an 60X sequencing coverage will be needed to detect 50% of the expected indels, and 1000X coverage will be needed to identify 90% of indels (Fig 6C). Importantly, instead of relying on next-generation sequencing, the model’s calculations can be used to design and prioritize the use of indel-specific assays that detect the presence of mutations in specific off-target sites with the highest model-predicted cleavage efficiencies.

### Optimal Experimental Conditions for Controlling (d)Cas9 Activity

The efficiency of Cas9-based cleavage and dCas9-based gene regulation depends on several factors, some controllable and others uncontrollable and host-specific. By manipulating the controllable factors, while accounting for the host-specific ones, on-target and off-target (d)Cas9 activity can be appropriately varied as desired. In particular, in the future, it may become necessary to tune the extent of dCas9-based transcriptional regulation to more precisely control gene expression levels. To aid in rational experimental design, we use the model to show how all the system parameters affect (d)Cas9 activity and to present general guidelines for achieving desired on-target and off-target activities.

First, we applied the model to calculate the dynamics of Cas9-based cleavage in actively growing cells, comparing several scenarios. The baseline model parameters are listed in S3 Table. Intuitively, DNA sites with additional mismatches have reduced cleavage efficiencies both at early and later time-points (Fig 7A). Perhaps less intuitively, increasing the guide RNA’s concentration by 10-fold beyond the baseline of 207 nM does not significantly increase steady-state cleavage efficiencies, but instead accelerates the cleavage process so that the steady-state condition is reached earlier. Further, if the organism’s cellular division rate increases by 2-fold, for example if the growth conditions or media are altered, then both the rates of cleavage and the steady-state cleavage efficiencies will drop by up to 2-fold. An increased growth rate has two general effects: it more quickly replenishes bound DNA sites with newly replicated ones, and it lowers the concentrations of Cas9 and guide RNA by dilution. Finally, and more substantially, carrying out genome mutagenesis in another organism with a 10-fold larger genome has a large slowing effect on Cas9 diffusion and overall cleavage activity, greatly reducing cleavage efficiencies at all DNA sites.

We next performed a sensitivity analysis on the model to examine how varying a system parameter affected cleavage at a single DNA site in an actively growing and dividing cell. If the DNA is fully complementary to the guide RNA sequence, its model-calculated minimum possible binding free energy will be ΔG_target_ = -9.9 kcal/mol, which yields a steady-state cleavage efficiency of 98% (Fig 7B). Consistent with our earlier examples, as Cas9’s binding free energy increases (lower affinity) above -9 kcal/mol, there will be significant drop in cleavage efficiency. DNA sites with ΔG_target_ > -4.9 kcal/mol will have <1% cleavage efficiencies. The concentration (or number) of guide RNA will also have a significant effect on cleavage efficiencies, but only when the guide RNA is a limiting substrate in the formation of the active Cas9:crRNA complex. As a result, when increasing the guide RNA concentration, cleavage efficiencies will rise until a critical threshold and thereafter there will be a plateau in cleavage efficiency (Fig 7B).

We next examined how these same parameters affected dCas9-based transcriptional regulation, and found similar relationships. The binding free energy between a guide RNA and its DNA site (ΔG_target_) controls both the dynamics and steady-state transcription rate of a dCas9-regulated promoter (Fig 7C). The binding free energy can be tuned by purposefully introducing mismatches into the guide RNA; within the linear regime, a 1.0 kcal/mol increase in ΔG_target_ will lower the binding occupancy of dCas9:crRNA by about 5-fold, which will increase a promoter’s transcription rate if dCas9 is utilized as a repressor (Fig 7D). The guide RNA concentration may also be controlled by employing environmentally-sensitive or inducible promoters. dCas9:crRNA’s binding occupancy at a particular DNA site depends sigmoidally on the crRNA expression level. There is a small range of crRNA expression levels where the largest change in dCas9:crRNA and promoter repression will take place. The addition of auxiliary crRNA binding sites will shift this sigmoidal curve rightwards. Large changes in binding occupancy also occur when the organism’s growth rate is increased or when gene regulation takes place in another organism with a larger genome (Fig 7C and 7D). Below, we discuss the implications of these parameter sensitivities when engineering dCas9-based genetic circuits.

## Discussion

We have developed the first mechanistic, quantitative model of CRISPR/Cas9 that encompasses the multi-step process responsible for Cas9-based genome editing and dCas9-based gene regulation. Our dynamical model holistically accounts for the kinetics of expression and formation of the active Cas9:crRNA complex, mass transfer by passive three-dimensional diffusion, genome-wide site selection according to the formation of R-loops at PAM-containing DNA sites, and the kinetics of irreversible site binding (Fig 1). We parameterized the model by combining both *in vitro* and *in vivo* measurements of (d)Cas9 activity (Table 1), arriving at a 11 parameter model (Table 2 and S2 Fig) that could explain how the concentrations of crRNA and Cas9 (Figs 2 and S3), DNA site supercoiling (Fig 3), canonical and non-canonical PAM sites (Table 3), and the thermodynamics of R-loop formation (Figs 4 and S4) all collectively control genome-wide (d)Cas9 activity. In particular, we provide newly obtained measurements showing that R-loop formation at adjacent crRNA binding sites has an anti-cooperative effect on dCas9-based gene regulation, which can be explained by positive supercoiling of the surrounding DNA and the destabilization of R-loop formation (Fig 3).

As part of our model-building, we found that once Cas9 binds to a target DNA site and begins to form an R-loop, it is far more likely to spontaneously dissociate than successfully form the R-loop and cleave the DNA site. Based on *in vitro* cleavage assay measurements (Fig 2), Cas9’s dissociation kinetic constant (k_d_) is 625-fold higher than its cleavage kinetic constant (k_c_), suggesting that hundreds of rounds of binding, melting, strand displacement, and abortive dissociation occur before cleavage takes place, which would be similar to the binding dynamics of RNA polymerase during transcriptional initiation (Table 2). Interestingly, coincident with this observation, a recent study utilized FRET to show that the rate of dCas9 binding is much faster and more indiscriminate than the rate of Cas9 cleavage, due to coupled changes (allostery) in Cas9 that only activates DNA cleavage under a restricted protein conformation [59]. There have also been recent measurements of Cas9 activity at off-target sites that use non-canonical PAMs, including NAG, NGA, NAA, NTG, NGC, NCG, and NGT [3,49,50], independent of our model-building process. Many of these alternative PAMs arise from a bulge or gap between a canonical PAM site and the guide RNA sequence [3,50]; through systematic comparisons, we determined the energetic penalties of these gaps and bulges on (d)Cas9’s binding affinity (Table 3). We also developed three different free energy models for R-loop formation, showing that the thermodynamics of R-loop formation cannot be predicted using existing measurements of RNA:DNA mismatch free energies (S5 Fig). Instead, we developed a 277 parameter empirical nearest-neighbor model and parameterized it using over 5000 measurements of (d)Cas9 activity (Fig 4). According to our model, the PAM-proximal 8 nucleotide seed region is responsible for up to 81% of (d)Cas9’s binding affinity and a single mismatch in this region lowers Cas9’s binding affinity by 14-fold. However, it is clear that the differences between *in vitro* and *in vivo* measurements have a confounding effect on (d)Cas9 R-loop formation and activity (Fig 4).

By carrying out genome-wide calculations on the lambdaphage and human genomes, mirroring recent experimental studies, we illustrated several physical principles governing (d)Cas9 activity that remain relevant regardless of model parameterization. First, Cas9 irreversibly binds to DNA sites in a hierarchical order, and its occupation of the highest affinity on-target sites causes its rate of binding to off-target sites to substantially increase (Fig 5). Therefore, both Cas9 concentration as well as incubation time are critical factors that control off-target activity as anecdotally observed in previous studies [60]. Second, off-target binding is highly heterogeneous across a genome; the binding occupancy at individual off-target DNA sites may be small, but the collective binding of Cas9 to all off-target sites is substantial. When Cas9-based genome editing is used as a therapeutic, the verification of off-target cleavage events will require very high sequencing coverage or rationally selected indel-specific detection assays (Fig 6). Third, our model explains why Cas9 off-target activity greatly varies across organisms. In bacteria, (d)Cas9 activity has been observed to be highly specific to its on-target sites [5,8] whereas, in human cells, next-generation sequencing has revealed thousands of off-target DNA cleavage events [5,29,42,50]. By modeling diffusion and genome-wide site specificity, we showed that the large increase in genome size, and not the difference in cell growth rate, is responsible for the observed increase in off-target activity. Importantly, it was necessary to identify and include both canonical and non-canonical PAM sites in our ensemble calculations to fully account for the breadth of off-target activity. These insights have the potential to greatly improve the predictive power of existing *in silico* target prediction methods [61,62] as recent observations have found that about 60% of off-target sites are not correctly predicted by existing bioinformatics models [50].

There are several practical steps that one can take to improve on-target Cas9 activity, while limiting off-target activity. First, the active Cas9:crRNA complex concentration controls the overall extent of off-target activity; if it’s high for a short period of time (10 nM for only 2 minutes and 100 nM for only 3 hours in bacterial and mammalian cells, respectively), then the rate of binding to off-target sites will not substantially rise as on-target sites have become occupied. During preparation of this article, two recent studies have implemented inducible Cas9 activity by expressing Cas9 using a doxycycline-inducible promoter [63] or by expressing a split version of Cas9 that uses rapamycin-inducible FRB domains to activate self-assembly [64]. Both approaches lowered the number of observed, off-target indel mutations. Second, during the design of guide RNA sequences, the search for off-target DNA sites must at least include both canonical and non-canonical PAMs (Table 3). A more thorough search would calculate Cas9’s binding affinity (ΔG_target_) across all accessible DNA sites, which would explicitly account for non-canonical PAMs as well as sequence- and distance-dependent mismatches. Third, several crRNAs may be designed and co-expressed to cleave the same genomic locus or to regulate the same promoter and thereby increase (d)Cas9 activity. According to our model, the occupancy of multiple Cas9:crRNA complexes at both on-target and off-target sites will be additive and independent so long as Cas9 expression is increased proportionally with the expression of additional crRNAs *and* when the on-target binding sites are separated by at least 200 bp to minimize the site-to-site effects of positive DNA supercoiling.

Finally, our modeling and experimental results have several implications when using dCas9-based gene regulation to engineer synthetic genetic circuits. First, extremely low crRNA expression levels are sufficient to form enough active dCas9:crRNA complexes to efficiently repress transcription because bacteria have small genomes and a low number of off-target sites. Further increases in crRNA expression had only a 3.7-fold change in transcriptional regulation as we observed in our reporter protein measurements (Fig 3). In other words, there are not enough DNA sites in bacterial cells to “sponge up” excess amounts of dCas9:crRNA complex. To increase an “inverter” circuit’s dynamic range, we showed that adding auxiliary on-target DNA sites on a high copy R6K plasmid will sequester dCas9:crRNA and shift the sigmoidal relationship between crRNA expression level and output promoter transcription rate. Adding either 2 or 4 auxiliary binding sites per plasmid (about 300 or 600 sites total) increased the circuit’s dynamic range by 27- or 11-fold. Second, mismatches can be purposefully introduced into on-target DNA sites to control binding occupancy, and therefore control transcriptional regulation. According to our model, a mismatch in the first 8 bp PAM-proximal region will (on average) increase ΔG_target_ by 0.78 kcal/mol and lower the binding occupancy of the dCas9:crRNA by 3.7-fold. Incorporating more mismatches will increase ΔG_target_ additively and decrease binding occupancy in a multiplicative manner. Third, when several crRNAs are expressed, they will competitively bind to dCas9 to form different dCas9:crRNA complexes, causing the increased expression of one crRNA to lower the concentration of another Cas9:crRNA complex. Such non-orthogonal relationships are generally undesired when engineering digital genetic circuits, and can be alleviated by expressing dCas9 in proportion to the total crRNA level. However, mutual dependence between dCas9:crRNA activities may be productively used to engineer analog signal processing circuits. Fourth, the effects of DNA supercoiling will have an impact on genetic circuit function. For example, computations using several input signals can be performed by co-regulating the same output promoter using different dCas9:crRNA complexes at adjacent crRNA binding sites. Even though the crRNA binding sites are adequately spaced apart to prevent steric interactions, site-to-site DNA supercoiling will inhibit the binding of one dCas9:crRNA when another has already bound, for example, by 11-fold when there are 4 nearby auxiliary sites. This anti-cooperative mechanism should be taken into account when engineering such “fan-in” genetic circuits.

## Materials and Methods

### Modeling the Expression and Formation of the Cas9:crRNA Complex

Mature crRNA guide strands can be expressed in two ways: transcription of a single chimeric synthetic guide RNA (sgRNA) that contains the 5' target recognition region, followed by a conserved Cas9-binding hairpin [43]; or transcription of a precrRNA array and a tracrRNA that form an RNA duplex that is subsequently processed by RNAse III into a mature crRNA [2,65]. As a key difference, the precrRNA can contain multiple target recognition sequences, each separated by a repetitive spacer sequence. The tracrRNA binds to these repetitive spacers and forms a double-stranded complex with precrRNA, becoming a target for RNAse III cleavage [2,65,66]. The resulting RNAse processing can generate multiple mature crRNAs from a single precrRNA. Cas9 may bind with the tracrRNA before landing on the precrRNA, and facilitate the tracrRNA:precrRNA hybridization [65]. After the mature crRNA is loaded into Cas9, an unidentified RNA exonuclease trims its 5' end, leaving a target recognition sequence of about 20 nucleotides [46,66]. When not bound to a crRNA, wild-type Cas9 remains in a structural conformation that inhibits its cleavage activity [67]. During the crRNA loading process, Cas9 undergoes a rotational shift that exposes a DNA binding channel, yielding an active Cas9:crRNA complex.

In our model, we first introduce the production rates of mature crRNA guide strands (r_crRNA_) and Cas9 proteins (r_Cas9_) as zero order reactions. These production rates can be varied by altering the DNA copy numbers or transcription rates of the precrRNA, sgRNA, or Cas9 as well as the translation rate of Cas9's mRNA [5,49]. We then employ mass action kinetics to describe the irreversible formation of an intermediate Cas9:crRNA complex, followed by an irreversible isomerization reaction that produces an active Cas9:crRNA complex. The rate of intermediate complex formation is quantified using a second order kinetic constant k_f_ and the isomerization reaction's rate is quantified using a first order kinetic constant k_I_. As first order reactions, the crRNA, Cas9, and intermediate Cas9:crRNA complex degrade or become diluted at a rate quantified by the kinetic constants δ_crRNA_, δ_Cas9_, and δ_Cas9:crRNA_. Finally, the rate of target binding for each active Cas9:crRNA complex is designated r_binding_, and will be derived below.

The resulting differential equations (Eqs 1–4) describe the dynamics of Cas9 and crRNA expression and active complex formation in terms of their molecular counts, assuming that the cell has a constant volume. For our first biophysical model of the CRISPR/Cas9 system, we have ignored the effects of stochastic gene expression as well as the effects of discrete cellular division. In addition, to account for the production of multiple crRNA guide strands with different sequences, we expanded the system of differential equations by an index *i* to describe their production, active complex formation, and rate of target binding. We assumed that all expressed crRNA guide strands bind equally well to Cas9, and form active complexes at the same rate, with the same kinetic parameters (k_f_ and k_I_). However, through competitive binding, the fraction of Cas9 bound to each crRNA guide strand will depend on the crRNAs' differing expression levels. The rates of Cas9-dependent cleavage will also differ across different crRNA guide strand sequences (index *i*) as well as different DNA site sequences (index *j*), designated by r_C [i,j]_.

$$\frac{{\text{dN}}_{\text{crRNA},\text{i}}}{\text{dt}}={\text{r}}_{\text{crRNA},\text{i}}-\delta_{\text{crRNA},\text{i}}{\text{N}}_{\text{crRNA},\text{i}}-{\text{k}}_{\text{f}}{\text{N}}_{\text{Cas}9}{\text{N}}_{\text{crRNA},\text{i}}$$ (1)

$$\frac{{\text{dN}}_{\text{Cas}9}}{\text{dt}}={\text{r}}_{\text{Cas}9}-\delta_{\text{Cas}9}{\text{N}}_{\text{Cas}9}-{\text{k}}_{\text{f}}{\text{N}}_{\text{Cas}9}\sum_{\text{j}}{\text{N}}_{\text{crRNA},\text{j}}$$ (2)

$$\frac{{\text{dN}}_{\text{intermediate},\text{i}}}{\text{dt}}={\text{k}}_{\text{f}}{\text{N}}_{\text{Cas}9}{\text{N}}_{\text{crRNA},\text{i}}-\delta_{\text{Cas}9:\text{crRNA}}{\text{N}}_{\text{intermediate},\text{i}}-{\text{k}}_{\text{I}}{\text{N}}_{\text{intermediate},\text{i}}$$ (3)

$$\frac{{\text{dN}}_{\text{Cas}9:\text{crRNA},\text{i}}}{\text{dt}}={\text{k}}_{\text{I}}{\text{N}}_{\text{intermediate},\text{i}}-\delta_{\text{Cas}9:\text{crRNA}}{\text{N}}_{\text{Cas}9:\text{crRNA},\text{i}}-\sum_{j}{\text{r}}_{\text{C}\;[\text{i},\text{j}]}$$ (4)

### Modeling Cas9's Random Walk to Determine Its Search Rate

Once formed, active Cas9:crRNA complexes do not undergo facilitated diffusion or hopping, but instead engage in three-dimensional molecular diffusion to search for DNA sites [38]. The rate of diffusion is governed by the diffusivity of the Cas9:crRNA complex (*D*), and also several host-specific factors, including the volume of the compartment (*V*) and the characteristic length between sites of production and binding (λ). Here, we assume that the cellular compartment is well-mixed such that the rate of *net* molar flow is zero, though the time required for a Cas9 protein to find a target DNA site depends on the rate of molecular diffusion. Accordingly, the rate of molecular diffusion for active Cas9:crRNA complexes using the i^th^ crRNA guide strand (*r*_*RW*,*i*_) will be proportional to its concentration [68]:

$${\text{r}}_{\text{RW},\text{i}}=\frac{{6\text{D}\mathrm{\lambda N}}_{\text{Cas}9:\text{crRNA},\text{i}}}{\text{V}}$$ (5)

Eq (5) is the molar flow rate, or contact rate, between active Cas9:crRNA complexes and all possible DNA sites inside the cell. We then use the sequences of the crRNA guide strand and the DNA site to calculate the probability that, once contact has been made, the active Cas9:crRNA complex binds to the DNA site and forms a stable Cas9:crRNA:DNA complex, called an R-loop. The rate of binding of the i^th^ Cas9:crRNA complex to the j^th^ DNA site is simply the product of the contact rate and the binding probability (*P*_*[i*,*j]*_):

$${\text{r}}_{\text{binding},[\text{i},\text{j}]}={\text{P}}_{\left[\text{i},\text{j}\right]}\;{\text{r}}_{\text{RW},\text{i}}$$ (6)

To calculate this binding probability, we assume that the pool of active Cas9:crRNA complexes have reached chemical equilibrium with the pool of both on-target and off-target DNA sites. This assumption is valid because the number of potential DNA sites is always much larger than the number of Cas9:crRNA complexes. In addition, when the Cas9:crRNA levels have reached steady-state conditions, the system will become ergodic. Accordingly, we derive a partition function in terms of the i^th^ active Cas9:crRNA complex's binding free energy to the j^th^ DNA site sequence (ΔG_target,[i,j]_) as well as the number of accessible DNA sites with the j^th^ sequence (*N*_*target*,*j*_). Here, our reference state is a DNA sequence that binds non-specifically to Cas9:crRNA with a zero binding free energy. As the total number of non-specific DNA binding sites, we use twice of the host's genome length *N*. The binding probability will follow a Boltzmann distribution, and we may use both the reference state and partition function as normalization factors to calculate the probability that the i^th^ Cas9:crRNA complex binds successfully to the j^th^ DNA site:

$${\text{P}}_{\left[\text{i},\text{j}\right]}=\frac{\frac{{\text{N}}_{\text{target},\text{j}}}{\text{N}}\text{exp}\left(-\frac{{\mathrm{\Delta G}}_{\text{target}[\text{i},\text{j}]}}{{\text{k}}_{\text{B}}\text{T}}\right)}{1+\sum_{\text{m}}\frac{{\text{N}}_{\text{target},\text{m}}}{\text{N}}\text{exp}\left(-\frac{{\mathrm{\Delta G}}_{\text{target}[\text{n},\text{m}]}}{{\text{k}}_{\text{B}}\text{T}}\right)}$$ (7)

Together, Eqs 6 and 7 provide a systematic approach for comparing the rates of binding for different crRNA sequences. Our next step was to develop a sequence-dependent free energy model to calculate and predict these binding rates for any crRNA guide strand sequence.

### A Free Energy Model for DNA Target Binding, R-Loop Formation, and Cleavage

The binding free energy of an active Cas9:crRNA complex to a particular DNA site controls its binding occupancy, and ultimately, its cleavage rate. Several interactions control the magnitude of this binding free energy, including the presence of a protospacer adjacent motif (PAM) site, the rate of R-loop formation during a multi-step exchange reaction, and the effects of supercoiling at the DNA site. Here, we employed thermodynamics to quantify the energetics of these interactions and developed a multi-term free energy model that calculates ΔG_target [i,j]_ for different crRNA guide strand sequences, DNA site sequences, canonical and non-canonical PAM sequences, and varying amounts of DNA site supercoiling. Altogether, the free energy model sums together the strengths of these interactions, according to:

$${\mathrm{\Delta G}}_{\text{target}[\text{i},\text{j}]}={\mathrm{\Delta G}}_{\text{PAM},\text{j}}+{\mathrm{\Delta \Delta G}}_{\text{exchange}[\text{i},\text{j}]}+{\mathrm{\Delta \Delta G}}_{\text{supercoiling},\text{j}}$$ (8)

Next, we describe the mechanism of R-loop formation and how these interactions' free energies are quantified.

After contacting a DNA site, a Cas9:crRNA complex recognizes and binds to the PAM sequence [35,69]. The canonical PAM site for the Cas9 from *Streptococcus pyogenes* is NGG, though additional non-canonical sequences have also been recognized [3,4,8,37]. The Cas9:crRNA complex then pulls apart the double-stranded DNA upstream of the PAM sequence, which is an energetically intensive process. Cas9 does not hydrolyze an energy-providing cofactor, such as ATP or GTP. Instead, its only significant source of external energy input originates from the binding interactions between the Cas9 protein and the PAM recognition sequence [38], which we designate as ΔG_PAM_. As we show below, the most canonical PAM recognition sequence has an apparent ΔG_PAM_ of about -9.5 kcal/mol, which is sufficient to pull apart four G:C or eight A:T DNA base pairings. Non-canonical PAM sequences have less energetically favorable interactions with Cas9, but can still support R-loop formation and cleavage [3,37,44,49].

The Cas9:crRNA complex continues to pull apart double-stranded DNA by performing an exchange reaction, allowing the crRNA guide strand to form RNA:DNA base pairings with its complementary DNA strand [38,44,70]. In step-wise transitions, each DNA base pair is pulled apart, and the corresponding nucleotide from the crRNA binds to form a Watson-Crick base pair, resulting in the formation of a DNA:Cas9:crRNA:DNA sandwich, called an R-loop. R-loop formation is directional and sequential, beginning at the PAM site, and proceeding upstream. Before the R-loop is completed, strand displacement can stall and reverse, resulting in Cas9:crRNA dissociation, whenever the DNA:DNA complex becomes more stable than the DNA:Cas9:crRNA:DNA complex. We designated this difference in stability as ΔΔG_exchange_; if ΔΔG_exchange_ becomes positive and large, the R-loop can not successfully form. To investigate whether Cas9 plays a role in target specificity, we then developed and parameterized two versions of a free energy model to calculate ΔΔG_exchange_ for a given crRNA and DNA site sequence, where the first model incorporates only nucleic acid interactions, while the second model accounts for both nucleic acid and Cas9-dependent interactions.

In the first model version, when the crRNA and DNA site are fully complementary, ΔΔG_exchange_ is governed by the difference in free energy between the RNA:DNA duplex and its corresponding DNA:DNA duplex. Interestingly, this difference is free energy is sequence-dependent; for example, the binding free energy of the dinucleotide base pair rAC:dGT is 1.0 kcal/mol more stable than dAC:dGT, while the binding free energy of rCG:dCG is 1.6 kcal/mol less stable than dCG:dCG [53]. These nearest-neighbor free energies are designated as ΔG^RNA:DNA^ and ΔG^DNA:DNA^, and may be calculated using previously developed free energy models that have been parameterized using calorimetry measurements [52,53,54,55]. Second, because of the sequential nature of R-loop formation, when the crRNA has non-complementary bases with the DNA site, the effect of the resulting mismatches will depend on their distance from the PAM site. For simplicitly, we introduce a position-dependent multiplicative weight *d*_*k*_ that modulates the impact of these free energy differences. *k* is location and varies from 0 to the crRNA guide strand's length; the value of d_1_ will be larger than d_20_.

Therefore, our first approach for calculating ΔΔG_exchange_ compares the thermodynamic stability of the i^th^ crRNA:DNA complex to the stability of the j^th^ DNA:DNA duplex, using the following expression:

$${\mathrm{\Delta \Delta G}}_{\text{exchange}[\text{i},\text{j}]}=\sum_{\text{k}}{\text{d}}_{\text{k}}\left[{\mathrm{\Delta G}}_{\text{k},\text{k}+1}^{\text{RNA}:\text{DNA}}-{\mathrm{\Delta G}}_{\text{k},\text{k}+1}^{\text{DNA}:\text{DNA}}\right]$$ (9)

where the summations proceed over the lengths of the crRNA:DNA and DNA:DNA sequences. Eq (9) has 21 unknown d_k_ parameter values and uses dinucleotide free energies that were previously parameterized in the absence of Cas9 [52,53,54,55].

However, it is possible that the Cas9 protein alters the stability of the R-loop in a sequence-specific fashion. To investigate this possibility, our second approach to calculating ΔΔG_exchange_ is to formulate an entirely empirical nearest-neighbor model, which enumerates all possible dinucleotide RNA:DNA duplexes and mismatches together with the distance-dependent coefficients, resulting in 277 unknown parameters. In the result section below, we determined these parameter values using thousands of experimental measurements of off-target and on-target Cas9 activity. Once parameterized, the following expression is used to calculate ΔΔG_exchange_ for any crRNA and DNA site sequence:

$${\mathrm{\Delta \Delta G}}_{\text{exchange}[\text{i},\text{j}]}=\sum_{\text{k}}{\text{d}}_{\text{k}}{\mathrm{\Delta \Delta G}}_{\text{k},\text{k}+1}^{\text{Cas}9:\text{crRNA}:\text{DNA}}$$ (10)

where the summation proceeds over the length of the crRNA:DNA sequence. In the results section, we systematically compared the accuracy of these two models to quantitatively determine Cas9's effect on DNA site specificity.

Next, we incorporated the effects of DNA site supercoiling into the model of Cas9:crRNA's binding energetics. Negative supercoiling, the untwisting of helical DNA, increases the stability of an R-loop by lowering the stability of the competing DNA:DNA complex [44]; however, there is a free energy input to form supercoiled DNA. When relaxed B-form helical DNA of length *n* is (un)twisted by 10σ turns, the change in free energy will be ΔG_supercoiling_ = 10*n*σ^2^k_b_T, where σ is the superhelical density, k_b_ is the Boltzmann constant, and T is temperature [71]. Due to the activity of topoisomerases and gyrases inside cells, the superhelical density of bacterial and human genomic DNA varies between σ = -0.02 and -0.1, depending on the location's distance from the origin of replication and its proximity to highly transcribed genes [72,73]. If a DNA site has already been negatively supercoiled by the host’s native enzymes, then a free energy input is not needed to stabilize the R-loop. However, if the DNA site is relaxed or positively supercoiled, then the additional free energy needed to untwist it will increase the dissociation rate of the Cas9:crRNA complex as it forms the R-loop. Accordingly, the dissociation kinetic constant of the Cas9:crRNA complex will depend on the degree of DNA site supercoiling according to

$${\text{k}}_{\text{d},\text{j}}={\text{k}}_{\text{d}}^{*}\text{exp}\left(-{\mathrm{\Delta G}}_{\text{supercoiling},\text{j}}/{\text{k}}_{\text{b}}\text{T}\right)$$ (11)

where we determine the free energy input needed to untwist the DNA site by comparing the superhelical density of an R-loop in its final state (σ_F_) with the initial superhelical density of the DNA site using (σ_I_) the expression.

$${\mathrm{\Delta G}}_{\text{supercoiling},\text{j}}=-{10\text{nk}}_{\text{b}}\text{T}\left(\sigma_{\text{F}}^{2}-\sigma_{\text{I}}^{2}\right)$$ (12)

The change in supercoiling energy in Eq 7 is a result of binding to a target from a non-specific site. Therefore, the energy term must be calculated based on the change in superhelical density of these targets (Eq 13). An average superhelical density of -0.06 for all nonspecific binding sites (σ_NS_) has been previously reported for *E*. *coli* genome [74,75].

$${\mathrm{\Delta \Delta G}}_{\text{supercoiling},\text{j}}=-{10\text{nk}}_{\text{b}}\text{T}\left(\sigma_{\text{I}}^{2}-\sigma_{\text{NS}}^{2}\right)$$ (13)

After the R-loop has formed, the DNA:Cas9:crRNA:DNA complex has the ability to cut the DNA strands, one at a time, typically at the third nucleotide upstream of the PAM site [4]. As measured by a time-course cleavage assay, an appreciable amount of nicked DNA accumulates before double-stranded DNA breaks are observed, indicating that Cas9’s endonuclease reaction is a slow, rate-limiting step. Unlike most enzymes, after Cas9 has doubly cut its DNA site, the Cas9:crRNA complex remains stably bound to the DNA site and does not have the ability to cleave DNA at another site [38]. This absence of turnover causes Cas9 to become a limiting reactant. However, before Cas9 has doubly cut its DNA site, optical trap pulling experiments have shown that the formation of the R-loop is reversible and that the DNA:Cas9:crRNA:DNA complex can dissociate [44]. In light of these two competing pathways, we derived an expression for the cleavage rate of the i^th^ Cas9:crRNA complex bound to the j^th^ DNA site:

$${\text{r}}_{\text{C}[\text{i},\text{j}]}=\frac{{\text{k}}_{\text{c}}}{{\text{k}}_{\text{c}}+{\text{k}}_{\text{d},\text{j}}}{\text{r}}_{\text{binding}[\text{i},\text{j}]}$$ (14)

where the rate of cleavage is controlled by a first-order kinetic constant k_C_ and the effects of DNA supercoiling on the dissociation kinetic constant, k_d_, are determined using Eq 11.

### Modeling the Genome-Wide Occupancies at On-Target and Off-Target DNA Sites

Finally, we calculate the total numbers of free, bound, and cut DNA sites over time by accounting for the production of DNA sites via DNA replication and their consumption by Cas9-based cleavage. Initially, the host organism begins with N_total,j_ copies of an accessible DNA site (type *j*). For chromosomally encoded DNA sites, N_total,j_ will vary between 0 and 2, depending on their distance from the chromosome's origin of replication and whether the site is located within accessible euchromatin or inaccessible heterochromatin. For plasmid-encoded DNA sites, N_total,j_ is the plasmid’s DNA copy number. After Cas9 binds and cleaves a DNA site, we assume that Cas9 remains bound to the site. After cleavage, the rate of DNA repair via homologous recombination or non-homologous end-joining will depend on several factors, for example, the host organism and the concentration of the repair DNA template. Here, we assume that the rate of DNA repair is proportional to the number of cut DNA sites. We also assume that, in actively growing cells, the replication rate of DNA sites is the cell’s division rate, designated as μ. Once a newly available DNA site has been replicated, it is distributed to daughter cells during division. Therefore, the net production rate of available DNA sites is the cell’s growth rate multiplied by the number of cleaved DNA sites, which is equivalent to μ(N_total,j_—N_target,j_),where N_target,j_ is the number of unbound DNA sites. Together, the rate of DNA replication and Cas9-dependent cleavage determines the total number of cut and uncut DNA sites within the organism, according to:

$$\frac{{\text{dN}}_{\text{target},\text{j}}}{\text{dt}}=\mu \left({\text{N}}_{\text{total},\text{j}}-{\text{N}}_{\text{target},\text{j}}\right)-\sum_{\text{i}}{\text{r}}_{\text{C}[\text{i},\text{j}]}$$ (15)

Altogether, for a genetic system that expresses η crRNAs in a host with ζ available DNA sites, the formally complete biophysical model of CRISPR/Cas9 consists of 3η + ζ (η+1) + 1 ordinary differential equations, which can be a large number. With further time-scale analysis that distinguishes between on-target and off-target DNA sites, there are several options for greatly reducing the number of partition function calculations and differential equations to determine the fraction of DNA sites that are free, bound, or cut. In one example, in early time periods, the low cleavage rates for the off-target DNA sites causes their differential equations to be well-approximated as linear, as compared to the highly coupled and non-linear differential equations for the on-target DNA sites. The analytical solutions to the differential equations for the off-target sites can then substituted into the numerical integration of the on-target DNA sites’ differential equations. In another example, determining the steady-state numbers of on-target and off-target DNA sites requires the solution of a system of multivariate quadratic polynomials, which can be efficiently computed using an iterative hybrid Krylov method [76]. With the availability of such analytical and numerical approximations, it is possible to solve the complete model using a mammalian genome without computational intractability, though an analysis to find the best approximation remains a topic for a future study.

### The Effects of Supercoiling on Adjacent DNA Sites

When a Cas9:crRNA complex binds to a DNA site, the formation of the R-loop will result in positive supercoiling of the surrounding DNA sites, due to conservation of the DNA linking number in the absence of topoisomerase or gyrase activity [77]. Positive supercoiling of DNA will alter the affinities of DNA-binding proteins, such as RNA polymerase [78] or other Cas9:crRNA complexes. These longer-range effects become important when crRNAs are designed to bind to several nearby on-target DNA sites, for example, when targeting two different DNA sites with a chimeric dCas9-FokI fusion [39], when inserting recombinant DNA between two nicked or doubly cleaved DNA sites, or when using dCas9 to regulate the transcription rate of a promoter using multi-input logic. Whenever multiple on-target DNA sites are adjacently located, we therefore modified the free energy model for ΔG_target_ to incorporate the site-to-site effects of supercoiling.

Consider multiple DNA sites located within a short segment of DNA surrounded by a type of fixed end, for example, between two active promoters, DNA replication origins, or other sites where DNA-binding proteins constrain DNA topology. When Cas9:crRNA binds to one of these DNA sites, the unwinding of the DNA site during R-loop formation increases the superhelical density of the remaining DNA segment by an amount Δσ (more positive), which depends on the lengths of the DNA site and the DNA segment. With the increase in supercoiling from σ_j_ to σ_j_ + Δσ (from negative to less negative), Cas9:crRNA will require an additional free energy input to bind to the remaining DNA sites within the segment and form an R-loop, according to Eq (12). As more DNA sites are bound by Cas9:crRNA, we assume that the linking number is conserved, yielding an increase in superhelical density from σ_j_ to σ_j_ + *c* Δσ for *c* bound DNA sites. Eventually, the free energy needed to stabilize the R-loop will become sufficiently large to prevent Cas9:crRNA from binding additional DNA sites within this DNA segment. According to our calculations below, Δσ is about 0.0065. To calculate these binding probabilities, we modified the partition function in Eq (7), accounting for the combinations of states where Cas9:crRNA has bound c adjacent DNA sites with their corresponding supercoiling-dependent energy penalties.

### Additional Model Considerations and Assumptions

There are additional factors, not included within this model, that can affect Cas9's ability to recognize and bind crRNAs as well as cleave DNA sites. Outside of the crRNA guide sequence, the tracrRNA and sgRNA form four stem loop structures that are responsible for recognizing and binding to Cas9 [43]. While the third and fourth stem loops are not essential for recognition, truncation of these structures did reduce the stability of the Cas9:crRNA complex. In another study, it was observed that truncated sgRNAs resulted in lower cleavage rates at both on-target and off-target DNA sites, which suggests that there were either fewer active Cas9:crRNA complexes or that active complexes had lower intrinsic cleavage activities [33]. Here, the biophysical model assumes that the tracrRNA and sgRNA fold into the wild-type structure. Further, while Cas9 can bind well to both single- and double-stranded DNA, its cleavage rate is significantly reduced when bound to single-stranded DNA or a truncated double-stranded DNA site [38]. The current biophysical model only considers double-stranded DNA sites within long contiguous DNA, such as plasmids and genomes.

Overall, the developed mechanistic model can estimate the probability of binding and cleavage for any Cas9 target DNA. In addition to degradation rate of all the involved molecules (δ_i_), the final model’s parameters are k_f_, k_I_ (complex formation step), ΔG_PAM_, ΔΔG_exchange_, ΔG_supercoiling_, k_d_, c (stabilizing target binding), and k_c_ (cleavage step). The input parameters are the exposure time (t) and the production rate of Cas9 (r_Cas9_) and crRNA (r_crRNA_). For a system containing 1 type of crRNA and N on- and off-targets, the concentrations of free Cas9, crRNA, intermediate complex, free active Cas9:crRNA complex and the targets are unknown, and can be calculated by solving N+4 ordinary differential equations (Eqs 1–4, 14 and 15) simultaneously. In the following sections, we have used multiple *in vivo* and *in vitro* measurements to estimate the model parameters in different conditions. A summary of the studies and the utilized data is provided in Table 1.

### Model Simulation and Parameterization Approach

Differential equations were numerically integrated using a variable-order, adaptive time-stepping stiff numerical solver (ode15s) in MATLAB. For comparison to experimental measurements, the relative errors between model solution and experimental measurements were calculated over the measurements' time interval or after a steady-state condition was reached. In Table 1, we summarize the several types of experimental measurements used to parameterize and validate the model, including the number of degrees of freedom and the number of data-points in each experimental data-set. To identify a narrow range of best-fit parameter values, model parameterization was performed by using either a simple simplex method (fminsearch) or a Levenberg-Marquardt method (lsqnonlin) in MATLAB to minimize the sum of squared relative errors, followed by a parameter sensitivity analysis and visual comparisons to more precisely identify best-fit model parameters.

### Strains and Plasmids

To validate model predictions, we constructed three plasmids that employ dCas9 to transcriptionally regulate expression of a reporter protein. The first plasmid expresses the YFP fluorescent protein reporter on a R6K vector using a KanR antibiotic marker. The YFP expression cassette contains a σ^70^ promoter (J23100), a synthetic ribosome binding site designed by the RBS Calculator [79,80,81], a codon-optimized YFP coding sequence, and an efficient transcriptional terminator [82]. A primary crRNA binding site is located within the promoter region with the sequence (5'—TATCGTTAAGGTTACTAGAG—3'). Where noted, between one to eight auxiliary crRNA binding sites with the same sequence as the primary crRNA binding site, each separated by 80 nucleotides of randomized DNA, were inserted downstream of the transcriptional terminator. To insert auxiliary binding sites, gBLOCK DNA fragments (Integrated DNA Technologies) were synthesized and assembled with a digested vector fragment using T4 ligation. The second plasmid constitutively expresses Cas9 and tracrRNA on a p15A vector using an AmpR antibiotic marker. Plasmid construction was performed by PCR-amplifying the Cas9 and tracrRNA expression cassettes from the pdCas9 plasmid [8] and assembling with a PCR-amplified p15A vector fragment using Gibson's method [83]. The third plasmid expresses the precrRNA using an IPTG-inducible P_tac_ promoter on a ColE1 vector using a CmR antibiotic marker, and was constructed by PCR-amplifying the precrRNA cassette from pdCas9 and assembling it with a PCR-amplified ColE1 fragment using Gibson's method. The precrRNA contains two BsaI sites flanking the protospacer region, which were utilized to insert new crRNA guide sequences into the precrRNA with digestion and ligation of annealed oligonucleotides. Cloned plasmids were verified by sequencing. The three plasmids were electroporated together into *E*. *coli* pir116, and selected on triple antibiotic agar plates.

### Growth and Measurements

Transformed strains were grown overnight at 37°C and 200 RPM in LB Miller supplemented with 10 μg/ml chloramphenicol, kanamycin, and ampicillin (Sigma-Aldrich). 5 μl of cultures were diluted into fresh selective media in a 96-well microplate, incubated, and shaken at 37°C in a TECAN M1000 spectrophotometer. Serial dilutions were performed twice to maintain cells in the exponential phase of growth for a 12 hour period. 10 μl samples were extracted after the second and third serial dilutions and added to 200 μl Phosphate buffered saline (PBS) supplemented with 2 mg/mL kanamycin for halting growth. Single-cell YFP fluorescence from at least 20,000 cells were recorded by an BD Fortessa flow cytometer. The average YFP expression level was determined by taking the average of the fluorescence distribution and subtracting the average auto-fluorescence of *E*. *coli* pir116.

## Supporting Information

S1 Fig Measured versus predicted DNA cleavage for the *in vitro* experiments by Sternberg et al. (PDF)

S2 Fig Sensitivity analysis of individual kinetic parameters. Each parameter was perturbed individually and model predictions were compared with the *in vitro* measurements by Sternberg et al. to calculate relative error.
(PDF)

S3 Fig Measured versus predicted DNA cleavage for short target DNA. Normalized cleaved DNA measurements (Circles) using 25 nM short DNA fragment are compared to normalized model-predicted amounts of cleaved DNA (lines). (A) without any model correction. (B) Correcting for the reduction in the number of potential Cas9 binding sites. (C) correcting for both the size change and the effect of change in supercoiling of the target site. The plateau in cleavage percentage is dictated by the concentration of Cas9:crRNA and is similar in all cases. However the calculated cleavage rate at each time point varies as a function of DNA content and supercoiling density of the targets.
(PDF)

S4 Fig Measured versus predicted dCas9 repression activity for the *in vivo* experiments. dCas9 was guided to target a YFP-driven promoter in *E*. *coli*. Inserting 1, 2, 4, or 8 auxiliary Cas9 binding sites altered Cas9 distribution among its binding sites and changed the YFP production, as recorded by flow cytometry. By considering the effects of DNA supercoiling as dCas9 binds to these sites, the developed model correctly predicts the effect of these auxiliary site on the binding occupancy of the promoter.
(PDF)

S5 Fig Quantifying the effect of crRNA:target mismatches using currently available RNA:DNA and DNA:DNA energy parameters. For each dataset (Table 1), a set of 21 positional weights were determined that minimized the error of model predictions. (A) Positional weights for mismatches at different locations of a target. (B) The calculated exchange energy for each base-pair as the difference between RNA:DNA and DNA:DNA energy parameters using available energy values. (C) Predictions versus measurements for ΔΔG_exchange_. Pearson correlation of 0.56 and 0.26 for dataset I and dataset II respectively
(PDF)

S1 Table Parameters used in the calculations on the λ-phage genome. (PDF)

S2 Table Parameters used in the calculations on the human genome. (PDF)

S3 Table Parameters used in the (d)Cas9 sensitivity analysis. (PDF)
