# Supplementary material for: A Biophysical Model of CRISPR/Cas9 Activity for Rational Design of Genome Editing and Gene Regulation
Source: PLoS Comput Biol. 2016 Jan 29;12(1):e1004724. doi: 10.1371/journal.pcbi.1004724 (PMC4732943; doi:10.1371/journal.pcbi.1004724)
Supplement: S1 Fig — (PDF) [file pcbi.1004724.s001.pdf]

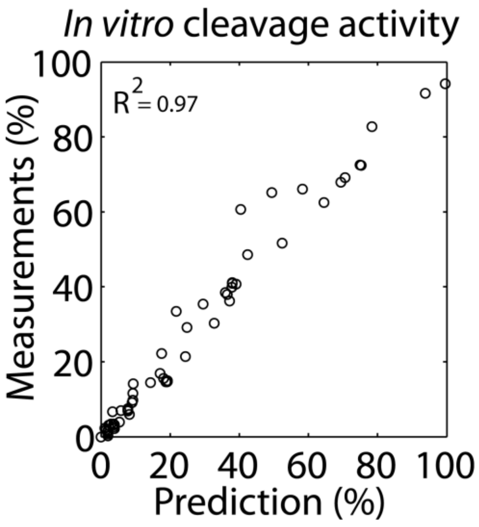

Supplementary Figure 1: Measured versus predicted DNA cleavage for the *in vitro* experiments by Sternberg et al.
