# Supplementary material for: A Biophysical Model of CRISPR/Cas9 Activity for Rational Design of Genome Editing and Gene Regulation
Source: PLoS Comput Biol. 2016 Jan 29;12(1):e1004724. doi: 10.1371/journal.pcbi.1004724 (PMC4732943; doi:10.1371/journal.pcbi.1004724)
Supplement: S2 Fig — Each parameter was perturbed individually and model predictions were compared with the in vitro measurements by Sternberg et al. to calculate relative error. (PDF) [file pcbi.1004724.s002.pdf]

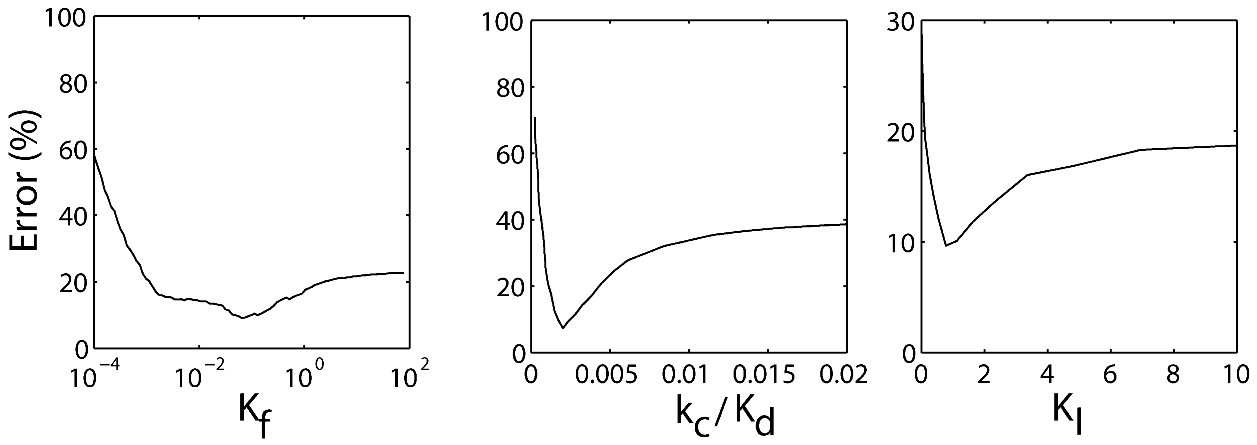

Supplementary Figure 2: Sensitivity analysis of individual kinetic parameters. Each parameter was perturbed individually and model predictions were compared with the *in vitro* measurements by Sternberg et al. to calculate relative error.
