# Supplementary material for: A Biophysical Model of CRISPR/Cas9 Activity for Rational Design of Genome Editing and Gene Regulation
Source: PLoS Comput Biol. 2016 Jan 29;12(1):e1004724. doi: 10.1371/journal.pcbi.1004724 (PMC4732943; doi:10.1371/journal.pcbi.1004724)
Supplement: S3 Fig — Normalized cleaved DNA measurements (Circles) using 25 nM short DNA fragment are compared to normalized model-predicted amounts of cleaved DNA (lines). (A) without any model correction. (B) Correcting for the reduction in the number of potential Cas9 binding sites. (C) correcting for both the size change and the effect of change in supercoiling of the target site. The plateau in cleavage percentage is dictated by the concentration of Cas9:crRNA and is similar in all cases. However the calculated cleavage rate at each time point varies as a function of DNA content and supercoiling density of the targets. (PDF) [file pcbi.1004724.s003.pdf]

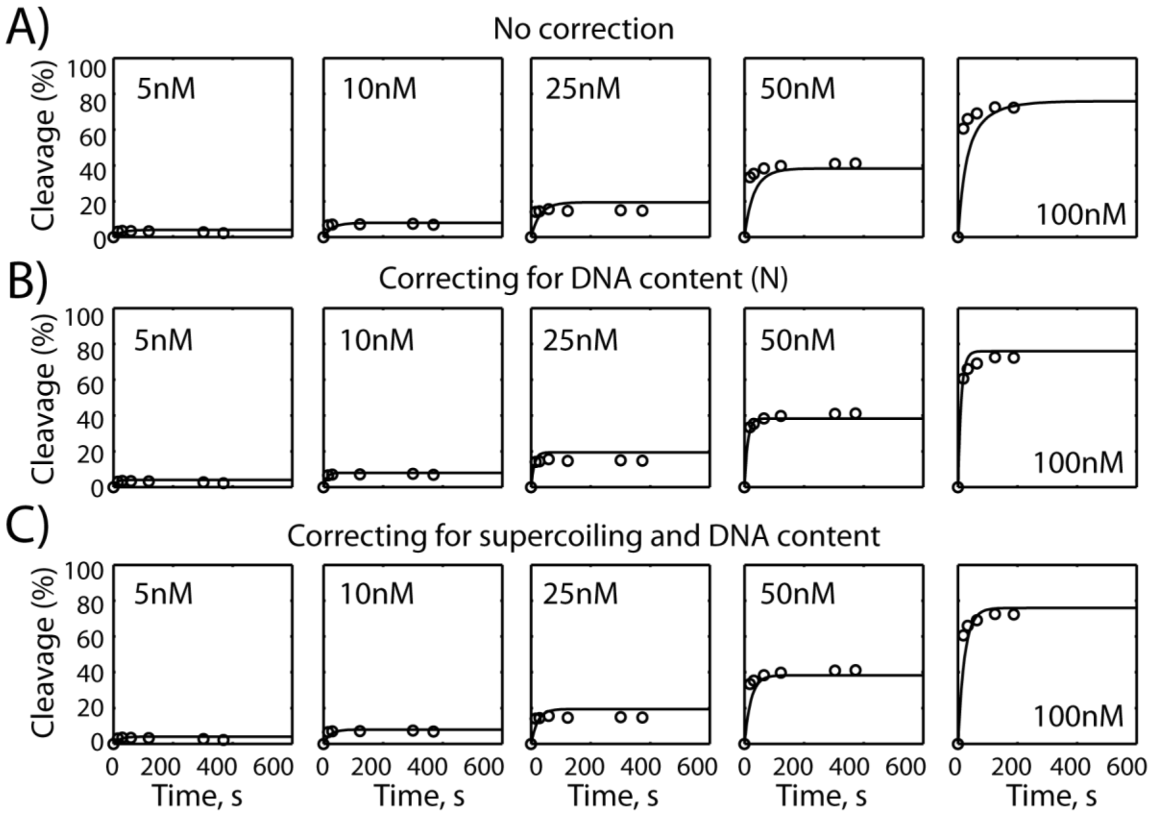

Supplementary Figure 3: Measured versus predicted DNA cleavage for short target DNA. Normalized cleaved DNA measurements (Circles) using 25 nM short DNA fragment are compared to normalized model-predicted amounts of cleaved DNA (lines). (A) without any model correction. (B) Correcting for the reduction in the number of potential Cas9 binding sites. (C) correcting for both the size change and the effect of change in supercoiling of the target site. The plateau in cleavage percentage is dictated by the concentration of Cas9:crRNA and is similar in all cases. However the calculated cleavage rate at each time point varies as a function of DNA content and supercoiling density of the targets.
