# Supplementary material for: A Biophysical Model of CRISPR/Cas9 Activity for Rational Design of Genome Editing and Gene Regulation
Source: PLoS Comput Biol. 2016 Jan 29;12(1):e1004724. doi: 10.1371/journal.pcbi.1004724 (PMC4732943; doi:10.1371/journal.pcbi.1004724)
Supplement: S4 Fig — dCas9 was guided to target a YFP-driven promoter in E. coli. Inserting 1, 2, 4, or 8 auxiliary Cas9 binding sites altered Cas9 distribution among its binding sites and changed the YFP production, as recorded by flow cytometry. By considering the effects of DNA supercoiling as dCas9 binds to these sites, the developed model correctly predicts the effect of these auxiliary site on the binding occupancy of the promoter. (PDF) [file pcbi.1004724.s004.pdf]

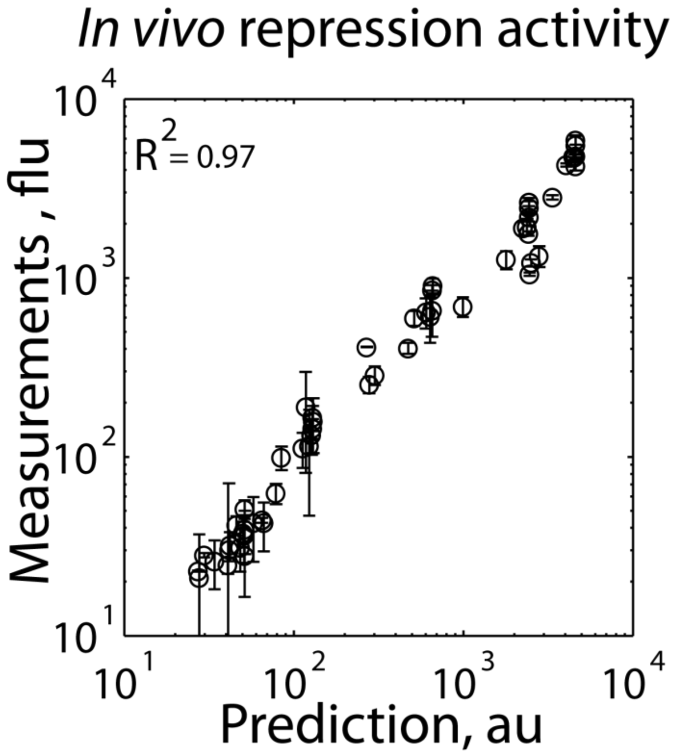

Supplementary Figure 4: Measured versus predicted dCas9 repression activity for the *in vivo* experiments. dCas9 was guided to target a YFP-driven promoter in *E. coli*. Inserting 1,2,4, or 8 auxiliary Cas9 binding sites altered Cas9 distribution among its binding sites and changed the YFP production, as recorded by flow cytometry. By considering the effects of DNA supercoiling as dCas9 binds to these sites, the developed model correctly predicts the effect of these auxiliary site on the binding occupancy of the promoter.
