# Supplementary material for: A Biophysical Model of CRISPR/Cas9 Activity for Rational Design of Genome Editing and Gene Regulation
Source: PLoS Comput Biol. 2016 Jan 29;12(1):e1004724. doi: 10.1371/journal.pcbi.1004724 (PMC4732943; doi:10.1371/journal.pcbi.1004724)
Supplement: S5 Fig — For each dataset (Table 1), a set of 21 positional weights were determined that minimized the error of model predictions. (A) Positional weights for mismatches at different locations of a target. (B) The calculated exchange energy for each base-pair as the difference between RNA:DNA and DNA:DNA energy parameters using available energy values. (C) Predictions versus measurements for ΔΔGexchange. Pearson correlation of 0.56 and 0.26 for dataset I and dataset II respectively (PDF) [file pcbi.1004724.s005.pdf]

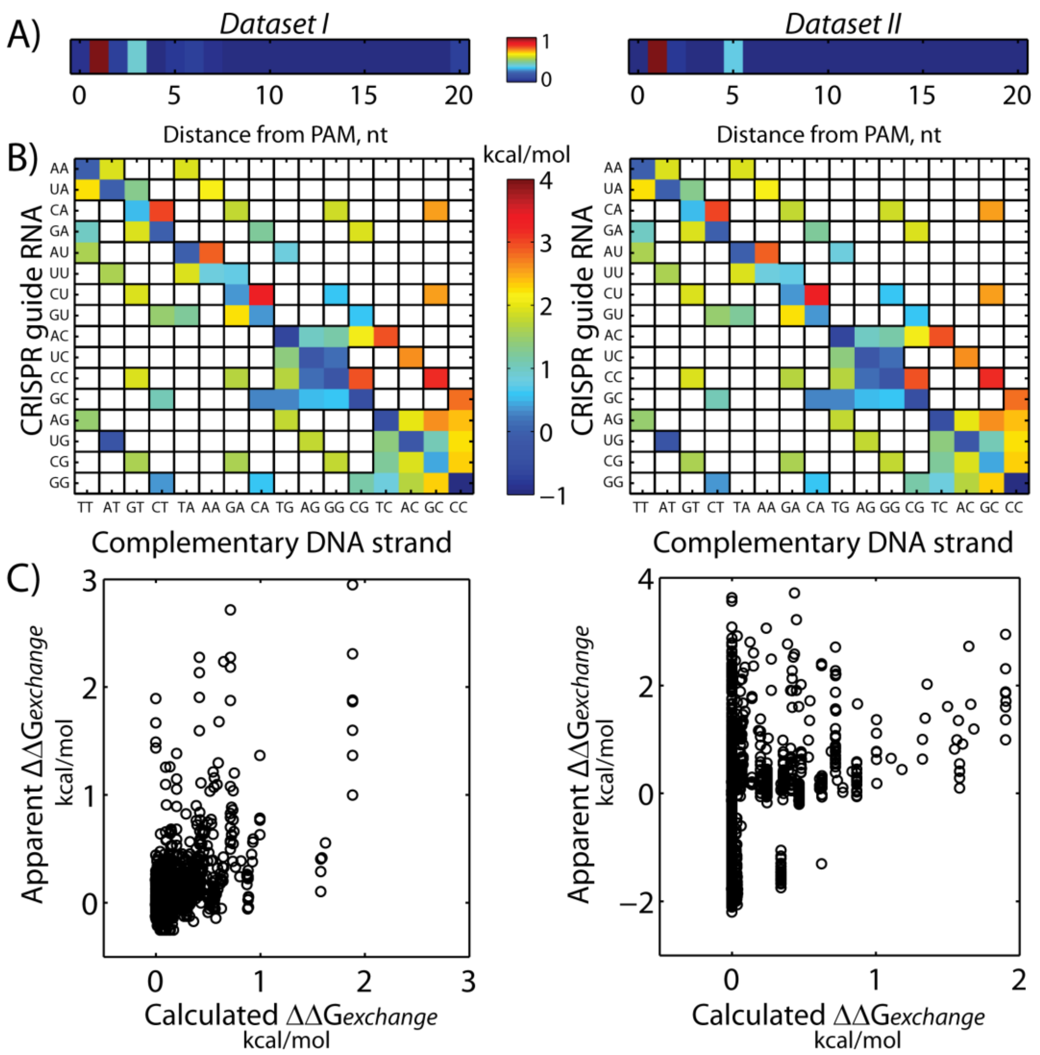

Supplementary Figure 5: Quantifying the effect of crRNA:target mismatches using currently available RNA:DNA and DNA:DNA energy parameters. For each dataset (Table 1), a set of 21 positional weights were determined that minimized the error of model predictions. (A) Positional weights for mismatches at different locations of a target. (B) The calculated exchange energy for each base-pair as the difference between RNA:DNA and DNA:DNA energy parameters using available energy values. (C) Predictions versus measurements for $\Delta\Delta G_{exchange}$. Pearson correlation of 0.56 and 0.26 for dataset I and dataset II, respectively.
