# Supplementary material for: A Biophysical Model of CRISPR/Cas9 Activity for Rational Design of Genome Editing and Gene Regulation
Source: PLoS Comput Biol. 2016 Jan 29;12(1):e1004724. doi: 10.1371/journal.pcbi.1004724 (PMC4732943; doi:10.1371/journal.pcbi.1004724)
Supplement: S3 Table — (PDF) [file pcbi.1004724.s008.pdf]

Supplementary Table 3: Parameters used in (d)Cas9 sensitivity analysis

Parameter	value	Unit
C_{cas9}	207	nM
C_{crRNA}	207	nM
**Doubling time**	20	hr
**N**	6.4×10^{9}	DNA sites
$\Delta\Delta G_{supercoiling}$	0	kcal/mol
$\Delta G_{on\text{-}target}$	-9.9	kcal/mol
$\Delta G_{single\text{-}mismatch}$	0.78	kcal/mol
